# NDUFS1 promotes malignant breast cancer behaviors through enhancement of mitochondrial respiration and PROX1/c-Myc signaling

**DOI:** 10.1016/j.isci.2026.117942

**Published:** 2026-10-19

**Authors:** Yen-Yun Wang, Pang-Yu Chen, Lester Uy Vinzons, Chih-Huang Tseng, Yuk-Kwan Chen, Stephen Chu-Sung Hu, Steven Lo, Ming-Feng Hou, Ching-Hu Wu, Yu-Chieh Chen, Shyng-Shiou F. Yuan

**Affiliations:** 1 School of Dentistry, College of Dental Medicine, Kaohsiung Medical University, Kaohsiung 807, Taiwan; 2 Department of Medical Research, Kaohsiung Medical University Hospital, Kaohsiung 807, Taiwan; 3 Drug Development and Value Creation Research Center, Kaohsiung Medical University, Kaohsiung 807, Taiwan; 4 Graduate Institute of Medicine, College of Medicine, Kaohsiung Medical University, Kaohsiung 807, Taiwan; 5 Division of Oral Pathology & Maxillofacial Radiology, Kaohsiung Medical University Hospital, Kaohsiung 807, Taiwan; 6 Department of Dermatology, College of Medicine, Kaohsiung Medical University, Kaohsiung 807, Taiwan; 7 Department of Dermatology, Kaohsiung Medical University Hospital, Kaohsiung 807, Taiwan; 8 Canniesburn Regional Plastic Surgery and Burns Unit, Glasgow G4 0SF, UK; 9 College of Medical, Veterinary and Life Sciences, University of Glasgow, Glasgow G12 8QQ, UK; 10 Division of Breast Oncology and Surgery, Department of Surgery, Kaohsiung Medical University, Kaohsiung 807, Taiwan; 11 Department of Obstetrics and Gynecology, Kaohsiung Medical University Hospital, Kaohsiung 807, Taiwan; 12 Translational Research Center, Kaohsiung Medical University Hospital, Kaohsiung 807, Taiwan; 13 Department of Biological Science and Technology, College of Biological Science and Technology, National Chiao Tung University, Hsinchu, Taiwan; 14 Center For Intelligent Drug Systems and Smart Bio-devices (IDS2B), National Yang Ming Chiao Tung University, Hsinchu 300, Taiwan

**Keywords:** breast cancer, NDUFS1, mitochondrial respiration, prox1, c-Myc

## Abstract

Cancer cells exploit oxidative phosphorylation (OXPHOS) for growth and metastasis. NDUFS1, the largest subunit of the electron transport chain complex I, is a promising therapeutic target in cancer but has a context-dependent role. Here, we elucidate the function of NDUFS1 in breast cancer. Elevated NDUFS1 expression was associated with advanced breast cancer stage and poor survival outcomes. *In vitro* experiments showed that NDUFS1 knockdown inhibited breast cancer cell proliferation, invasion, and stemness-associated properties, whereas NDUFS1 overexpression enhanced these phenotypes. Moreover, NDUFS1 directly regulated mitochondrial respiratory function in breast cancer cells and increased c-Myc expression via PROX1. In a mouse model of breast cancer, NDUFS1 knockdown in injected mammary carcinoma cells or OXPHOS inhibition decreased tumor growth and PROX1 and c-Myc expression. Our study highlights the role of the NDUFS1-PROX1-c-Myc axis in breast cancer progression, paving the way for new therapies targeting breast cancer mitochondrial OXPHOS.

## Highlights

• High NDUFS1 expression is correlated with advanced breast cancer and poor survival
• NDUFS1 drives breast cancer cell growth, invasion, and stemness-related properties
• NDUFS1 regulates PROX1/c-Myc signaling by modulating mitochondrial respiration
• Mitochondrial complex I inhibition suppresses breast tumor growth

## Graphical abstract

**Figure:**
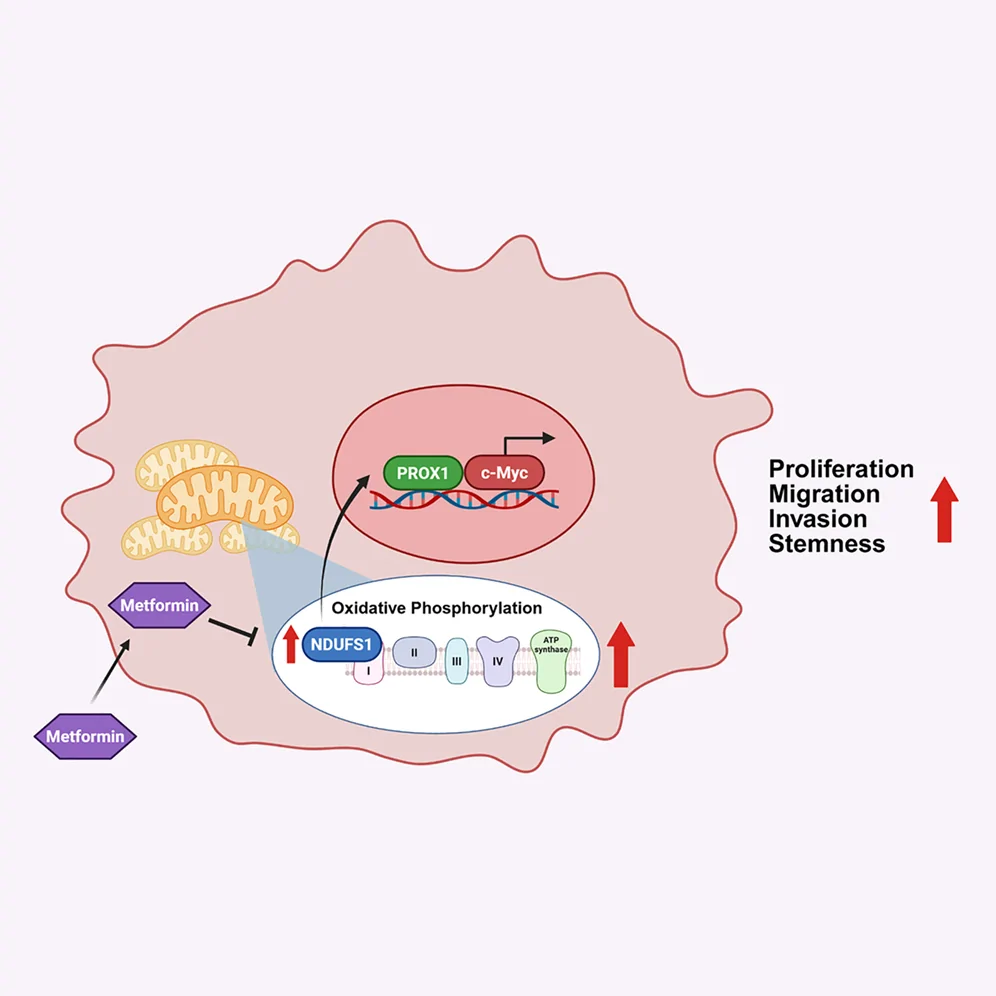

## Teaser

Biological sciences; Molecular mechanism of gene regulation; Cancer systems biology; Cancer

## Introduction

Metabolic reprogramming—particularly in oxygen sensing and energy metabolism^1^^,^^2^—is a hallmark of cancer cells, ensuring sustained cell growth and proliferation in the face of a harsh tumor microenvironment.^3^^,^^4^ Mitochondria are responsible for processes such as the tricarboxylic acid cycle, oxidative phosphorylation (OXPHOS), fatty acid oxidation, and signal exchange with other cell compartments to adjust to fluctuating metabolic demands.^5^ One of the key metabolic phenotypes responsible for cancer cell energy generation is the preferential dependence on glycolysis rather than OXPHOS, even with normal oxygen concentrations—commonly known as the Warburg effect—which was initially thought to be related to impairment of mitochondrial OXPHOS. However, recent studies challenge this assumption and indicate that mitochondrial OXPHOS, involving the electron transport chain (ETC) complexes I-IV and adenosine triphosphate (ATP) synthase (complex V), is largely intact and, in some instances, upregulated in cancers.^6^^,^^7^ A meta-analysis examining normal and cancer cell lines indicated that OXPHOS contributes to 80% of relative ATP production in normal cells and 83% in cancer cells.^8^ Inhibition of OXPHOS has also shown therapeutic efficacy in several cancer subtypes.^9^

Different cancers rely on mitochondrial metabolism, specifically OXPHOS, for tumor growth, metastasis, and therapy resistance.^10^^,^^11^^,^^12^ Pancreatic cancer cells surviving oncogene ablation have characteristics similar to cancer stem cells and utilize OXPHOS for survival.^13^ Acute myeloid leukemia with IDH mutation exhibits elevated mitochondrial oxidative metabolism, including an increase in ETC complex I activity.^14^ Enhanced OXPHOS activity was also detected in osteosarcoma tumors, with OXPHOS inhibitors greatly suppressing osteosarcoma cell survival.^15^ Breast cancer also relies on mitochondrial OXPHOS in a cell type- and context-dependent manner.^16^^,^^17^^,^^18^ Breast cancer of basal-like subtype with frequent RB1 loss and p53 disruption exhibits elevated mitochondrial metabolism.^19^ Breast cancer cells display higher complex IV activity compared to adjacent stromal and normal ductal epithelial cells.^20^ In an orthotopic mouse model of breast cancer, OXPHOS-deficient breast cancer cells did not form primary tumors, while breast cancer cells with very low levels of OXPHOS formed primary tumors and lung metastases.^21^ Following neoadjuvant chemotherapy of triple-negative breast cancer cells, surviving breast cancer cells exhibited mitochondrial fusion and increased OXPHOS activity.^22^

One of the most widely studied components of mitochondrial OXPHOS in cancer is the NADH:ubiquinone oxidoreductase core subunit S1 (NDUFS1), which is the largest subunit of the ETC complex I. NDUFS1 plays an essential role in complex I function, and mutations in the gene have been found to result in a reduced amount and activity of complex I,^23^^,^^24^ decreased stability of the complex I N-module,^25^ and increased susceptibility of the mitochondria to reactive oxygen species (ROS) damage.^24^ Notably, studies show a significant—albeit context-dependent—association between NDUFS1 expression and cancer progression. On one hand, decreased expression of NDUFS1 is associated with poor prognosis in non-small cell lung cancer^26^ and clear-cell renal cell carcinoma,^27^ radioresistance in colorectal cancer,^28^ and malignant progression in gastric cancer^29^ and lung adenocarcinoma.^30^ On the other hand, NDUFS1 has been found to be essential in PHB2-mediated promotion of colorectal cancer tumorigenesis^31^ as well as colorectal cancer cell survival in acidic conditions.^32^ Elevated NDUFS1 expression is also highly correlated with prostate cancer incidence and poor recurrence-free survival.^33^

Given the contrasting roles of NDUFS1 in different cancer contexts, it is imperative to clarify its effects in breast cancer in order to exploit its potential as a biomarker and a therapeutic target. Some efforts have been made in this regard, with one study linking NDUFS1 mutation to high pathogenicity in breast cancer,^34^ and another study showing that NDUFS1 upregulation promotes breast cancer cell proliferation.^35^ In this study, we further investigate the role of NDUFS1 in breast cancer progression *in vitro* and *in vivo* and provide clinical evidence of its potential in determining patient outcome. Additionally, we delineate a signaling pathway that links NDUFS1 to breast cancer cell proliferation, invasion, and stemness-associated phenotypes. Through these approaches, we elucidate the critical role of NDUFS1 and mitochondrial respiration in driving breast cancer malignancy and identify potential targets in breast cancer therapy.

## Results

### Elevated NDUFS1 is correlated with breast cancer progression and poor patient outcomes

The link between NDUFS1 expression and the clinicopathological features and clinical outcomes of breast cancer was first established using data from online databases and collected tissue samples and patient data from Kaohsiung Medical University Hospital (KMUH), Taiwan. From the Oncomine database, the expression level of NDUFS1 mRNA was found to be significantly higher in breast cancer tissues than in normal breast tissues (Figure 1A). This increased NDUFS1 gene expression was also observed in breast tumor tissues categorized according to breast cancer subtype based on The Cancer Genome Atlas (TCGA) and the Genotype-Tissue Expression (GTEx) data in the harmonized TCGA-TARGET-GTEx dataset (Figure 1B). To evaluate the protein expression of NDUFS1 in breast cancer, immunohistochemical analysis was performed on breast cancer tissues and normal breast tissues obtained from KMUH. Our data showed an elevated level of NDUFS1 expression in breast cancer tissues compared with normal tissues, as well as a higher NDUFS1 expression in later-stage breast cancer tissues (Figure 1C). Moreover, Kaplan-Meier survival analysis using publicly available breast cancer microarray datasets (Kaplan-Meier Plotter) showed that patients with high expression levels of NDUFS1 had significantly poorer recurrence-free survival and overall survival (Figure 1D). This is supported by Kaplan-Meier analysis of overall survival using the TCGA Breast Invasive Carcinoma (TCGA-BRCA) dataset from the Genomic Data Commons (GDC) (Figure 1E). Multivariable Cox proportional hazards analysis of the GDC TCGA-BRCA dataset using either age, tumor stage, and breast cancer subtype, or age, TNM (primary tumor/regional lymph node/distant metastasis) stage, and receptor status as covariates confirmed that higher expression of NDUFS1 was independently associated with worse overall survival in patients with breast cancer (Table S1). Further correlation analysis of NDUFS1 protein expression and clinicopathological characteristics of breast cancer from the KMUH patient data showed a significant positive association between NDUFS1 and breast cancer stage, tumor size (T stage), and lymph node (LN) metastasis (Table 1).

**Figure 1:**
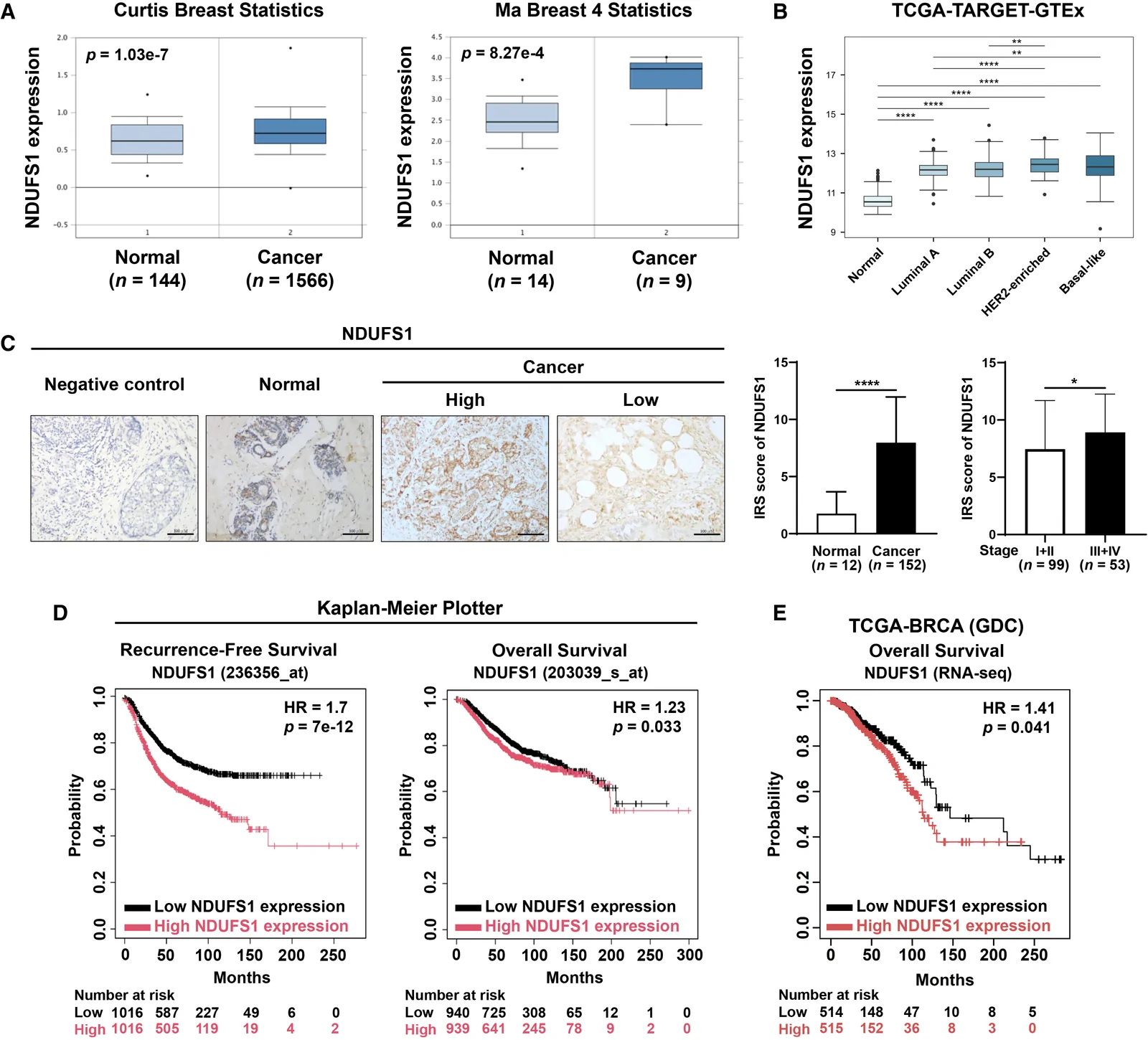
Elevated NDUFS1 is positively correlated with breast cancer progression and poor patient outcomes (A) Comparison of NDUFS1 mRNA expression between normal breast tissues and breast cancer tissues using the Oncomine platform (https://www.oncomine.org). (B) Comparison of NDUFS1 mRNA expression between normal breast tissues and subtype-categorized breast cancer tissues from the TCGA-TARGET-GTEx dataset (https://xenabrowser.net/) (normal: *n* = 178; luminal A: *n* = 420; luminal B: *n* = 192; HER2-enriched: *n* = 66; basal-like: *n* = 139). (C) Protein expression of NDUFS1 in breast cancer tissues and normal breast tissues, assessed using the immunoreactive score (IRS) method. Data are presented as mean ± SD. (D) Kaplan-Meier graph of recurrence-free survival and overall survival from the Kaplan-Meier Plotter (https://kmplot.com/analysis/), using publicly available breast cancer microarray datasets, stratified according to NDUFS1 mRNA expression. (E) Kaplan-Meier graph of overall survival of patients with breast cancer from the TCGA-BRCA (GDC) dataset, stratified according to NDUFS1 mRNA expression (also see Table S1). ∗, *p* < 0.05; ∗∗, *p* < 0.01; and ∗∗∗∗, *p* < 0.0001. See also Table S1.

**Table 1:** The association of NDUFS1 expression and clinicopathological characteristics of KMUH patients with breast cancer using logistic regression

Variable	NDUFS1		*p*-value	crude OR (95% CI)	*p*-value	adj OR (95% CI)
	Low	High				
	N (%)	N (%)				
Total	26 (17.1)	126 (82.9)	–	–	–	–
Age (years)	52.77±10.30	52.33±10.65	0.85	–	–	–

**Stage**						

I +II	24 (92.3)	75 (59.5)	0.0004	1.00	–	–
III+IV	2 (7.7)	51 (40.5)	–	8.16 (2.28-52.16)	–	–

**Graded**						

I	2 (7.7)	14 (11.1)	0.59	1.00	0.15	1.00
II+III	24 (92.3)	112 (88.9)	–	0.67 (0.10-2.60)	–	0.29 (0.05-1.57)

**BMI (kg/m**^**2**^**)**						

<24	17 (65.4)	66 (52.4)	0.22	1.00	0.22	1.00
≥24	9 (34.6)	60 (47.6)	–	1.72 (0.73-4.30)	–	1.89 (0.67-5.63)

**T stage**						

T1	19 (73.1)	46 (36.5)	0.0006	1.00	0.02	1.00
T2-T4	7 (26.9)	80 (63.5)	–	4.72 (1.92-12.87)	–	3.36 (1.17-9.63)

**LN metastasis**						

N0	20 (76.9)	39 (30.9)	<.0001	1.00	0.0007	1.00
N1-3	6 (23.1)	87 (69.1)	–	7.44 (2.92-21.66)	–	6.34 (2.17-18.48)

**ER status**						

Positive	18 (69.2)	78 (61.9)	0.48	1.00	0.96	1.00
Negative	8 (30.8)	48 (38.1)	–	1.38 (0.57-3.60)	–	0.96 (0.20-4.62)

**PR status**						

Positive	16 (61.5)	68 (54.0)	0.48	1.00	0.61	1.00
Negative	10 (38.5)	58 (46.8)	–	1.36 (0.58-3.33)	–	1.50 (0.32-7.03)

**HER2 status**						

Positive	17 (65.4)	81 (64.3)	0.92	1.00	0.93	1.00
Negative	9 (34.6)	45 (35.7)	–	1.05 (0.44-2.64)	–	0.96 (0.35-2.63)

### NDUFS1 promotes breast cancer cell proliferation, invasion, and stemness-associated phenotypes

Endogenous protein expression of NDUFS1 varied in six human breast cancer cell lines tested, namely, ZR-75-1, T-47D, MCF-7, BT-549, Hs 578T, and MDA-MB-231 cells (Figure S1A). We proceeded with further experiments on MCF-7 and MDA-MB-231 cells to model luminal A (ER-positive) and triple-negative breast cancer, respectively, with varying levels of NDUFS1 expression. NDUFS1 protein expression in MDA-MB-231 and MCF-7 cells was downregulated using shRNA clones #1 and #2, which showed better knockdown efficiency compared with four other clones (Figure S1B).

We used these NDUFS1-knockdown breast cancer cells (Figure S1C) as well as NDUFS1-overexpressing breast cancer cells (Figure S1D) to investigate the role of NDUFS1 in promoting breast cancer malignant phenotypes. Based on XTT assay, knockdown of NDUFS1 significantly decreased MDA-MB-231 and MCF-7 cell proliferation (Figure 2A), whereas overexpression of NDUFS1 significantly increased MDA-MB-231 and MCF-7 cell proliferation (Figure 2B). Similarly, NDUFS1 downregulation significantly suppressed the migration and invasion of both MDA-MB-231 and MCF-7 cells, while NDUFS1 upregulation significantly enhanced such behaviors based on Transwell assays (Figures 2C–2F).

**Figure 2:**
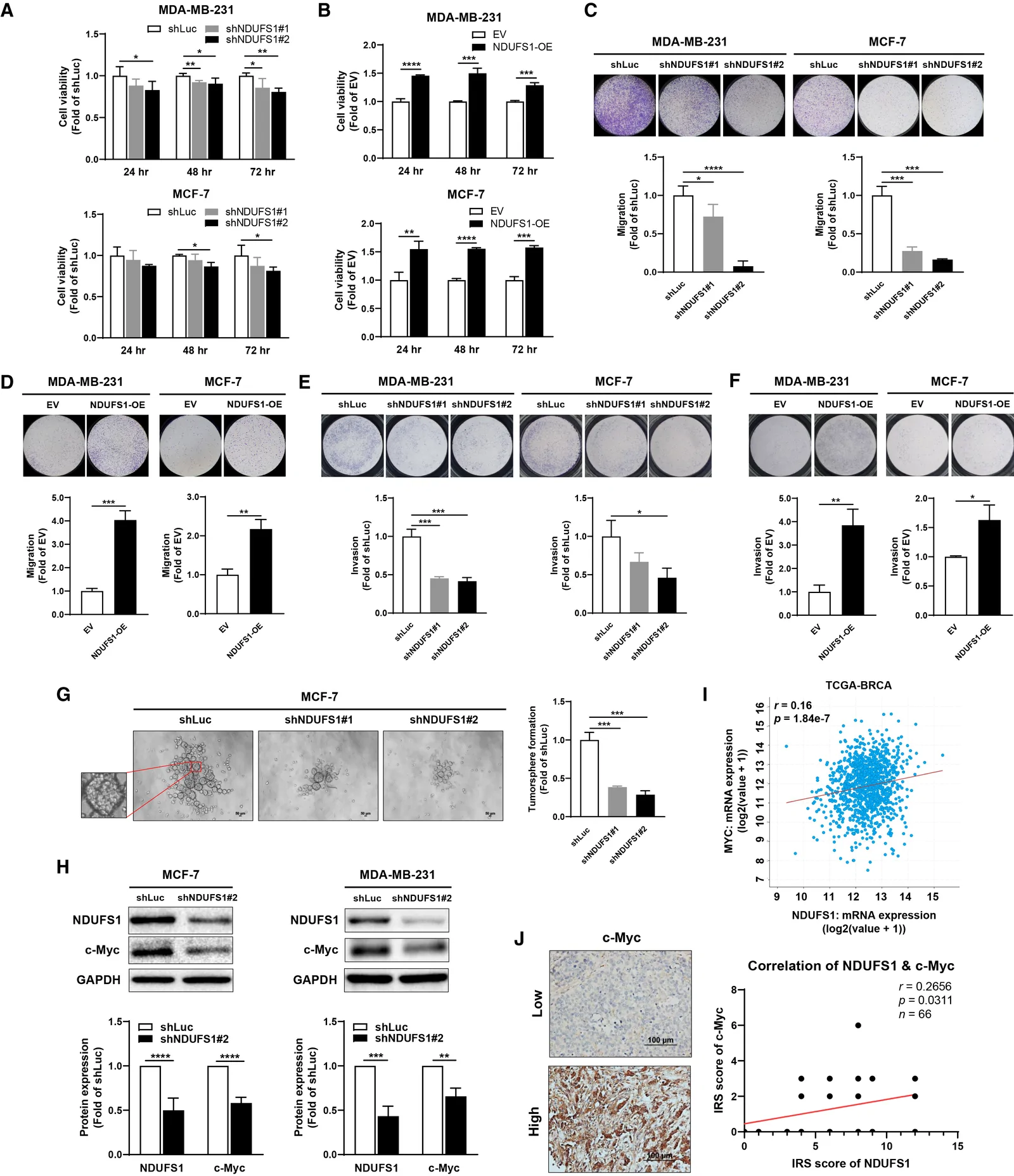
NDUFS1 promotes breast cancer cell proliferation, invasion, and stemness-associated phenotypes (A and B) Cell viability of MDA-MB-231 and MCF-7 cells at different time points after NDUFS1 knockdown (A) and overexpression (B), as determined by the XTT assay. (C and D) Migration behavior of MDA-MB-231 and MCF-7 cells after NDUFS1 knockdown (C) and overexpression (D), as determined by the Transwell assay. (E and F) Invasion behavior of MDA-MB-231 and MCF-7 cells after NDUFS1 knockdown (E) and overexpression (F), as determined by the Transwell assay. (G) Tumorsphere formation of MCF-7 cells after NDUFS1 knockdown. (H) Western blot analysis of c-Myc protein expression in MCF-7 and MDA-MB-231 cells after NDUFS1 knockdown. (I) Correlation between NDUFS1 and MYC mRNA expression in the TCGA-BRCA dataset. (J) Correlation between NDUFS1 and c-Myc protein expression in breast tumor tissues, as determined from the immunoreactive scores (IRS). Data are presented as mean ± SD from three independent experiments. ∗, *p* < 0.05; ∗∗, *p* < 0.01; ∗∗∗, *p* < 0.001; and ∗∗∗∗, *p* < 0.0001. shLuc, knockdown of firefly luciferase using shRNA; shNDUFS1#1, knockdown of human NDUFS1 using shRNA clone 1; shNDUFS1#2, knockdown of human NDUFS1 using shRNA clone 2; EV, empty vector; NDUFS1-OE, overexpression of NDUFS1. See also Figures S1 and S2.

To characterize the stemness-associated phenotypes, we utilized tumorsphere formation assay^36^^,^^37^ and western blot analysis of stemness-related markers. We observed that knockdown of NDUFS1 in MCF-7 cells reduced tumorsphere formation (Figure 2G) and significantly suppressed the expression of stemness-associated factors KLF4, c-Myc, and SOX2 (Figure S2A). In particular, NDUFS1 downregulation in both MCF-7 and MDA-MB-231 cells significantly decreased the expression of c-Myc (Figure 2H), an oncogene known for regulating cell stemness, growth, and proliferation.^38^^,^^39^ Conversely, NDUFS1 overexpression significantly upregulated c-Myc expression in both cell lines (Figures S2B and S2C).

Additionally, we explored the clinical association between NDUFS1 and c-Myc and found a positive correlation between NDUFS1 and MYC mRNA expression in patient breast tumor tissues using the TCGA-BRCA dataset (Figure 2I). We further employed immunohistochemical staining of patient breast tumor tissues and confirmed a positive correlation between NDUFS1 expression and c-Myc expression (Figure 2J). Taken together, these data suggest that NDUFS1 promotes malignant phenotypes and stemness-associated growth in breast cancer cells.

### NDUFS1 promotes malignant breast cancer cell behaviors via enhancement of mitochondrial respiration

The association between NDUFS1 and stemness-related phenotypes (Figure 2) suggests the involvement of mitochondrial respiration in oncogenesis. Thus, we further investigated the effect of NDUFS1 regulation on mitochondrial respiration using an extracellular oxygen consumption assay and the Seahorse XF Cell Mito Stress Test. Using the oxygen consumption assay, we found that knockdown of NDUFS1 decreased the basal oxygen consumption and oxygen consumption rate (OCR) in MDA-MB-231 and MCF-7 cells (Figure 3A), indicating impaired mitochondrial respiratory function, while overexpression of NDUFS1 increased both quantities (Figure 3B). Further OCR measurements using Seahorse mitochondrial modulators showed that NDUFS1 knockdown downregulated mitochondrial respiratory parameters: basal OCR, ATP production, maximal respiration, and spare respiratory capacity (Figure 3C), while NDUFS1 overexpression upregulated basal OCR and ATP production (Figure S3A). Notably, NDUFS1 knockdown in MDA-MB-231 and MCF-7 cells also decreased the extracellular acidification rate (ECAR) (Figures S3B and S3C), indicating that impairment of mitochondrial respiration did not lead to a compensatory increase in glycolysis in the breast cancer cells.

**Figure 3:**
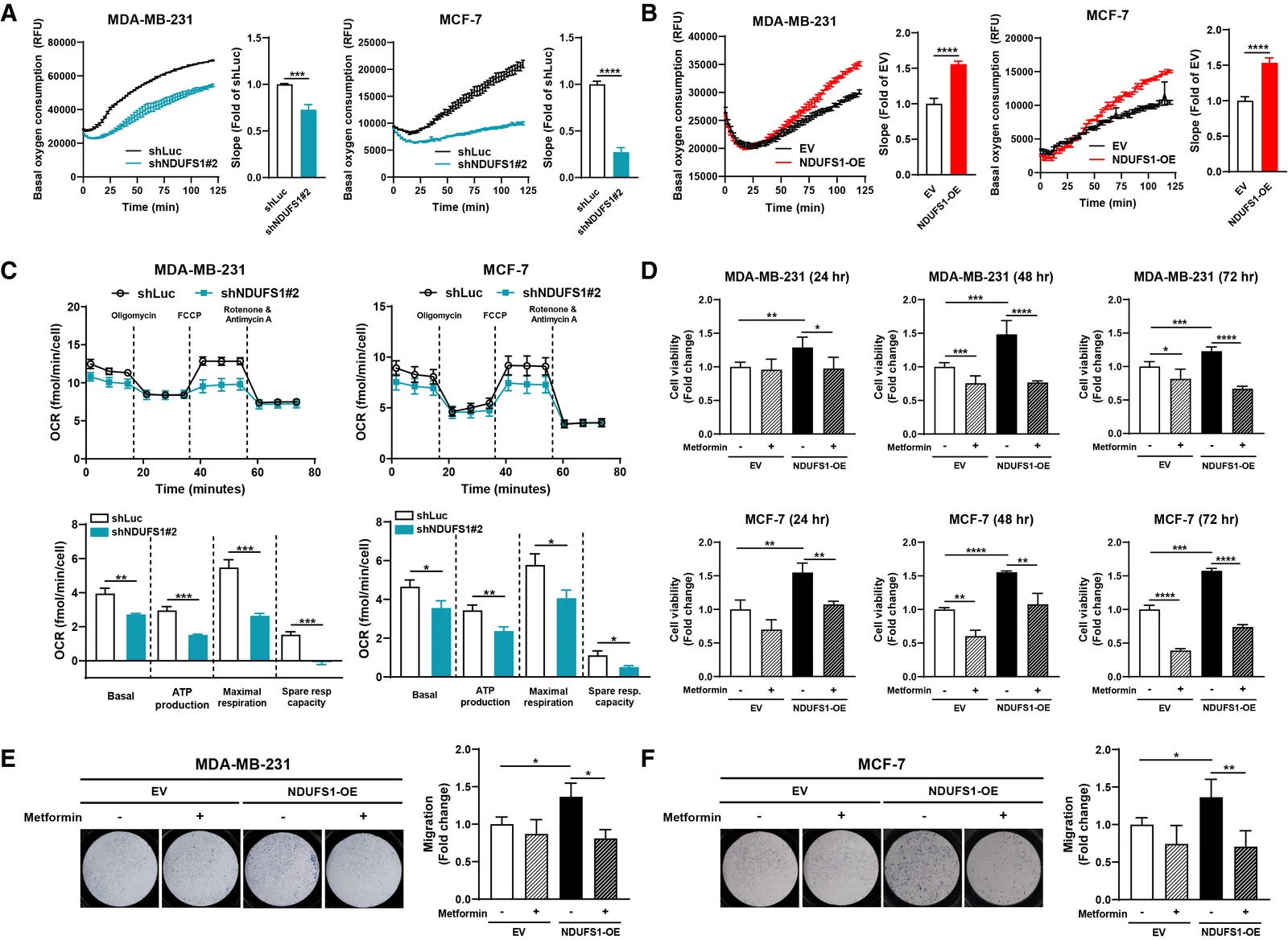
NDUFS1 promotes malignant breast cancer cell behaviors via enhancement of mitochondrial respiration (A and B) Basal oxygen consumption of MDA-MB-231 and MCF-7 cells after NDUFS1 knockdown (A) and overexpression (B), with the slope (ΔRFU/Δmin) being the oxygen consumption rate (OCR). (C) Mitochondrial respiratory parameters in MDA-MB-231 and MCF-7 cells after NDUFS1 knockdown, as measured by OCR using an Agilent Seahorse XFe24 Analyzer via sequential delivery of the indicated mitochondrial modulators. (D) Cell viability of MDA-MB-231 and MCF-7 cells with or without NDUFS1 overexpression after treatment with metformin (30 mM) for 24, 48, or 72 hours, as determined by XTT assay. Metformin was added one day after cell seeding. (E and F) Migration of MDA-MB-231 (E) and MCF-7 (F) cells with or without NDUFS1 overexpression after treatment with metformin (30 mM), as determined by Transwell assay. Metformin was added on the day of cell seeding. Data are presented as mean ± SD from three independent experiments. ∗, *p* < 0.05; ∗∗, *p* < 0.01; ∗∗∗, *p* < 0.001; and ∗∗∗∗, *p* < 0.0001. shLuc, knockdown of firefly luciferase using shRNA; shNDUFS1#2, knockdown of human NDUFS1 using shRNA clone 2; EV, empty vector; NDUFS1-OE, overexpression of NDUFS1. See also Figure S3.

After ascertaining the effect of NDUFS1 regulation on mitochondrial respiration, we further examined the role of mitochondrial respiration in NDUFS1-associated breast cancer cell aggressiveness using the mitochondrial complex I inhibitor metformin. We found that metformin treatment, at a concentration that does not affect cancer cell viability,^40^ reversed the NDUFS1-induced increase in MDA-MB-231 and MCF-7 cell proliferation as well as decreased cell proliferation in the vector-control cells following prolonged culture (Figure 3D). Moreover, metformin also suppressed the increased migration of MDA-MB-231 and MCF-7 cells induced by NDUFS1 overexpression (Figures 3E and 3F). These results indicate that NDUFS1 indeed mediates its effects on breast cancer cell malignant behaviors through the modulation of mitochondrial respiration.

### NDUFS1 regulates PROX1/c-Myc/p21 signaling through mitochondrial respiration

To identify the signaling pathway involved in the NDUFS1-driven malignant phenotype of breast cancer cells, we employed bulk RNAseq of MDA-MB-231 cells with or without NDUFS1 knockdown. Using overlap analysis of genes from three gene ontology clusters—migration, stemness, and proliferation—we identified three genes of interest, namely, PROX1, MEF2C, and WNT5A, which were downregulated after NDUFS1 knockdown based on both RNA-seq (Figure 4A) and real-time quantitative PCR (Figure S4). We further analyzed the association of these genes and their respective proteins with NDUFS1 expression as well as their clinical significance in breast cancer. Western blot analysis showed that PROX1 protein expression significantly decreased or increased as NDUFS1 was knocked down or overexpressed, respectively (Figure 4B), while Wnt-5a did not show a significant increase when NDUFS1 was overexpressed in MDA-MB-231 cells (Figure S5A). TCGA-BRCA analysis revealed that NDUFS1 expression was positively correlated with PROX1 expression (Figure 4C), which was further confirmed by immunohistochemical staining of breast tumor tissues (Figure 4D). Kaplan-Meier survival analysis using the Kaplan-Meier Plotter also showed that patients with high PROX1 expression exhibited a trend toward low recurrence-free survival (*p* = 0.074; Figure S5B). On the other hand, while TCGA-BRCA dataset analysis showed a positive correlation between NDUFS1 mRNA expression and MEF2C and WNT5A mRNA expression (Figure S5C), Kaplan-Meier survival analysis showed that patients with high levels of MEF2C and WNT5A expression had significantly better recurrence-free survival (Figure S5D), in contrast to NDUFS1 (Figure 1D). These results point to an important role of PROX1 in NDUFS1-induced breast cancer progression.

**Figure 4:**
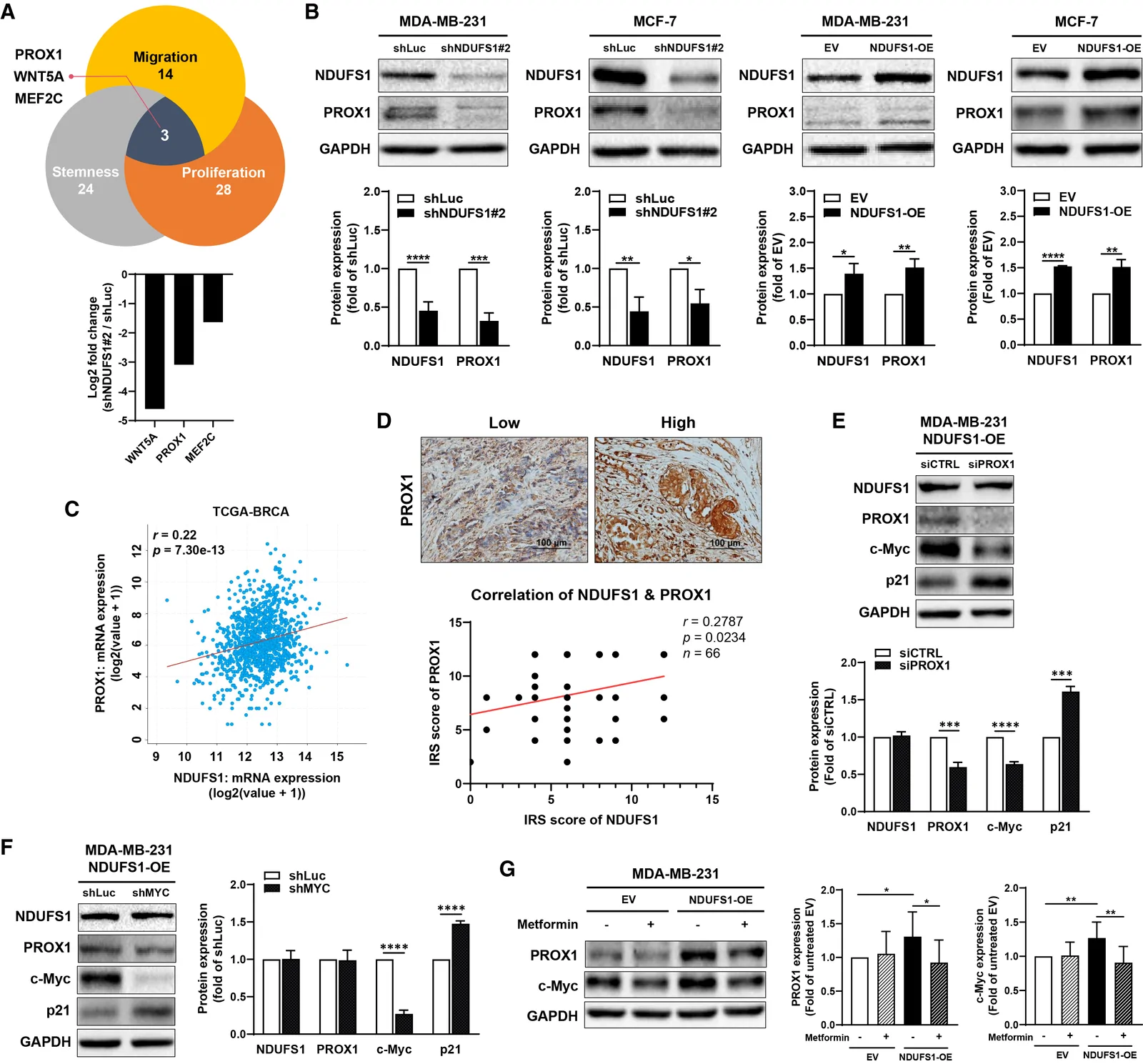
NDUFS1 regulates PROX1/c-Myc/p21 signaling through mitochondrial respiration (A) Genes involved in cell proliferation, stemness, and migration based on bulk RNA sequencing analysis of NDUFS1-knockdown MDA-MB-231 cells. The fold changes of the shared genes—PROX1, WNT5A, and MEF2C—are shown. (B) Western blot analysis of NDUFS1 and PROX1 protein expression in MDA-MB-231 and MCF-7 cells after NDUFS1 knockdown and overexpression. (C) Correlation between NDUFS1 and PROX1 mRNA expression in the TCGA-BRCA dataset. (D) Correlation between NDUFS1 and PROX1 protein expression in breast tumor tissues, as determined from the immunoreactive scores (IRS). (E and F) Western blot analysis of NDUFS1, PROX1, c-Myc, and p21 protein expression in NDUFS1-overexpressing MDA-MB-231 cells after knockdown of PROX1 (E) or MYC (F) via siRNA or shRNA, respectively. (G) Western blot analysis of PROX1 and c-Myc protein expression in MDA-MB-231 cells with or without NDUFS1 overexpression after metformin (30 mM) treatment for 24 hours. Data are presented as mean ± SD from three independent experiments. ∗, *p* < 0.05; ∗∗, *p* < 0.01; ∗∗∗, *p* < 0.001; and ∗∗∗∗, *p* < 0.0001. shLuc, knockdown of firefly luciferase using shRNA; shNDUFS1#2, knockdown of human NDUFS1 using shRNA clone 2; EV, empty vector; NDUFS1-OE, overexpression of NDUFS1; siCTRL, control knockdown using non-targeting siRNA; siPROX1, knockdown of PROX1 using siRNA; shMYC, knockdown of MYC using shRNA. See also Figures S4–S6.

We then investigated the effect of PROX1 in regulating the expression of the stemness-associated factor c-Myc, which we found to be directly modulated by NDUFS1 as well (Figure 2H). Knockdown of PROX1 by siRNA led to a reduced expression of c-Myc (Figure 4E), while downregulation of c-Myc by shRNA did not affect the expression of PROX1 (Figure 4F), indicating that PROX1 is an upstream regulator of c-Myc expression. This suggests that NDUFS1 controls the expression of c-Myc via PROX1. In addition, we found that knockdown of either PROX1 or c-Myc resulted in an increase in the expression of the cell cycle inhibitor p21 (WAF1/CIP1) (Figures 4E and 4F), consistent with a previous study showing that c-Myc suppresses p21 expression.^41^ Taken together, these results confirm the presence of an NDUFS1-PROX1-c-Myc-p21 signaling axis in breast cancer.

The NDUFS1-PROX1-c-Myc-p21 signaling axis points to the important role of mitochondrial respiration in PROX1 and c-Myc expression. To investigate whether OXPHOS-targeting pharmacologic perturbation can affect this signaling pathway, we treated parental and NDUFS1-overexpressing MDA-MB-231 cells with metformin. Metformin treatment reversed the increase in PROX1 and c-Myc expression induced by NDUFS1 overexpression without decreasing PROX1 and c-Myc levels in the vector-control cells (Figure 4G). This result may be due in part to the ability of metformin to downregulate NDUFS1 expression in NDUFS1-overexpressing MDA-MB-231 cells but not in parental MDA-MB-231 cells (Figure S6). These findings suggest an NDUFS1-level-dependent effect of OXPHOS-targeting drugs on the NDUFS1-PROX1-c-Myc-p21 signaling axis.

### Mitochondrial complex I inhibition suppresses orthotopic breast tumor growth and tumoral expression of PROX1 and c-Myc *in vivo*

The *in vivo* effect of NDUFS1 on breast tumor growth was evaluated using a syngeneic BALB/c mouse model and luciferase-expressing 4T1 mouse breast cancer cells. The efficiency of Ndufs1 knockdown in 4T1 cells was first confirmed by western blot analysis (Figures S7A and S7B). Similar to the human breast cancer cell lines, Ndufs1 knockdown in 4T1 cells significantly decreased cell proliferation, migration, invasion, and tumorsphere formation (Figures 5A–5D), with a concomitant decrease in the expression of the stemness-associated factor c-Myc (Figures 5E and S7C). Furthermore, Ndufs1 knockdown significantly inhibited the expression of PROX1 (Figure 5E) and increased the expression of p21 (Figure S7D), without markedly altering MEF2C and Wnt-5a expression levels (Figure S7E). 4T1 cells with Ndufs1 knockdown were injected into the left fourth mammary fat pad of each mouse, and then the tumor volume was measured weekly, with parallel monitoring of bioluminescence signals using an IVIS 50 *in vivo* imaging system. The mice were sacrificed when the tumor volume of the control group exceeded 1000 mm^3^, in accordance with the KMU IACUC guidelines.

**Figure 5:**
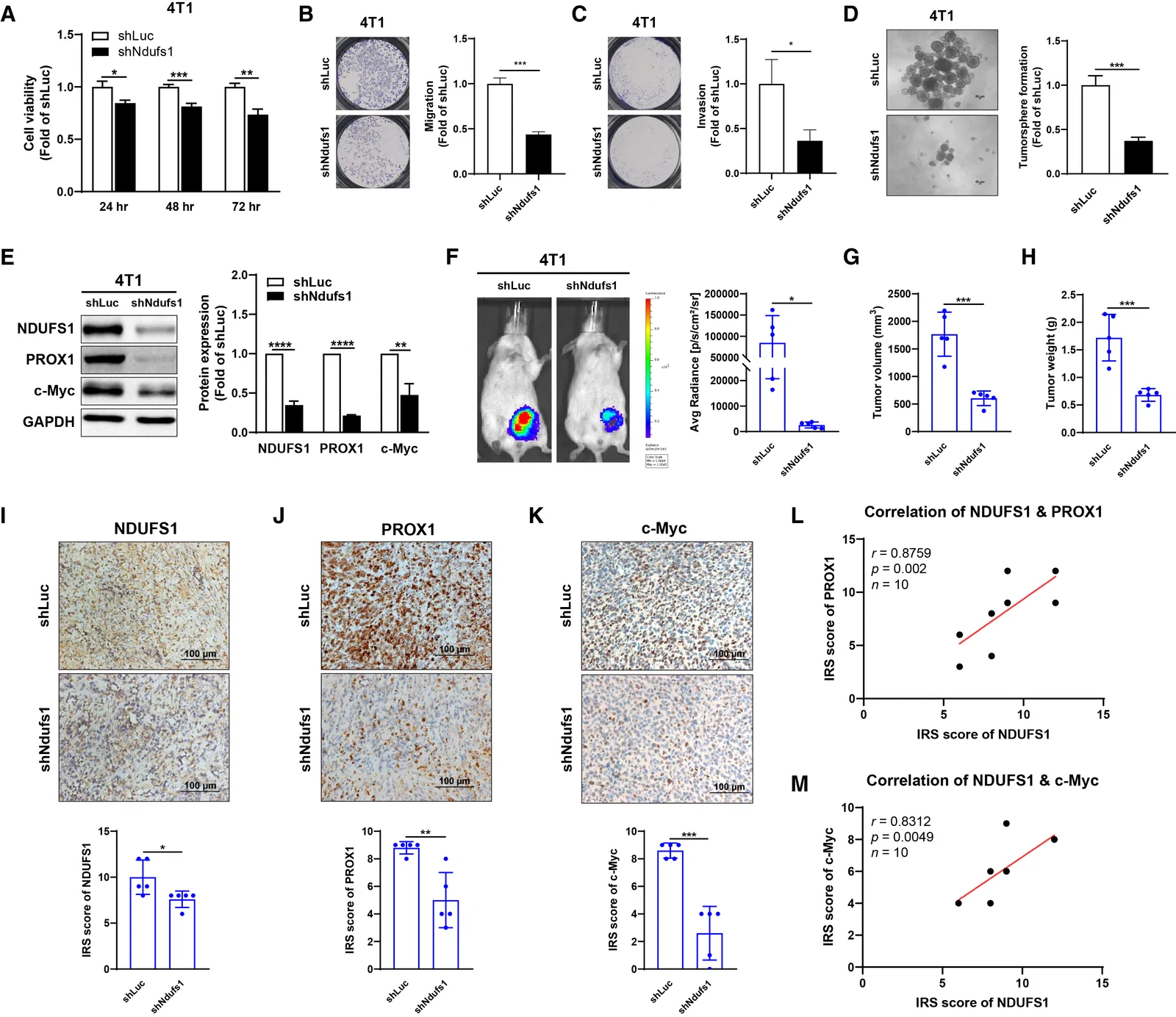
NDUFS1 promotes orthotopic breast tumor growth and tumoral expression of PROX1 and c-Myc *in vivo* (A) Cell viability of 4T1 cells at different time points after Ndufs1 knockdown, as determined by XTT assay. (B and C) Migration (B) and invasion (C) behaviors of 4T1 cells after Ndufs1 knockdown, as determined by Transwell assays. (D) Tumorsphere formation of 4T1 cells after Ndufs1 knockdown. (E) Western blot analysis of NDUFS1, PROX1, and c-Myc protein expression in 4T1 cells after Ndufs1 knockdown. (F) *In vivo* photon flux density emitted by luciferase-expressing 4T1 cells with or without Ndufs1 knockdown in BALB/c mice, detected and photographed by an IVIS 50 imaging system at the endpoint of the experiment. (G and H) Volume (G) and weight (H) of orthotopic tumors collected at the endpoint of the experiment. (I–K) Protein expression of NDUFS1 (I), PROX1 (J), and c-Myc (K) in the orthotopic tumors, assessed using the immunoreactive score (IRS) method. (L and M) Correlation of NDUFS1 expression with PROX1 (L) and c-Myc (M) expression in the orthotopic tumors, as determined from the IRS. Data are presented as mean ± SD; ∗, *p* < 0.05; ∗∗, *p* < 0.01; ∗∗∗, *p* < 0.001; and ∗∗∗∗, *p* < 0.0001. shLuc, knockdown of firefly luciferase using shRNA; shNdufs1, knockdown of mouse Ndufs1 using shRNA clone D3. See also Figure S7.

At the end of the experiment, the Ndufs1-knockdown group demonstrated significantly lower bioluminescence signals (Figure 5F), tumor volume (Figure 5G), and tumor weight (Figure 5H). No obvious metastases were detected by bioluminescence imaging, which may be due to an immune response toward the xenogeneic luciferase reporter protein in the 4T1 cells.^42^ Further immunohistochemical analysis of the tumor tissues showed that the expression of NDUFS1, PROX1, and c-Myc was significantly decreased in the Ndufs1-knockdown group (Figures 5I–5K). In addition, NDUFS1 expression in the tumor tissues was positively correlated with PROX1 and c-Myc expression (Figures 5L and 5M).

We then investigated the effectiveness of the mitochondrial complex I inhibitors metformin and rotenone in suppressing NDUFS1-mediated tumor growth *in vivo*. Metformin or rotenone was injected intraperitoneally five times per week for four weeks. At the end of the experiment, the metformin- and rotenone-treated shLuc groups showed significantly lower bioluminescence signals (Figure 6A), tumor volume (Figure 6B), and tumor weight (Figure 6C), which were comparable to those of the Ndufs1-knockdown group. Furthermore, immunohistochemical analysis of the orthotopic tumors showed that the expression of NDUFS1, PROX1, and c-Myc was significantly lower in the metformin- and rotenone-treated shLuc groups, closely mirroring the results of the Ndufs1-knockdown group (Figures 6D and 6E). These results show that mitochondrial OXPHOS inhibitors suppress the growth of NDUFS1-expressing breast tumors *in vivo* by downregulating key proteins in the NDUFS1-PROX1-c-Myc-p21 signaling axis.

**Figure 6:**
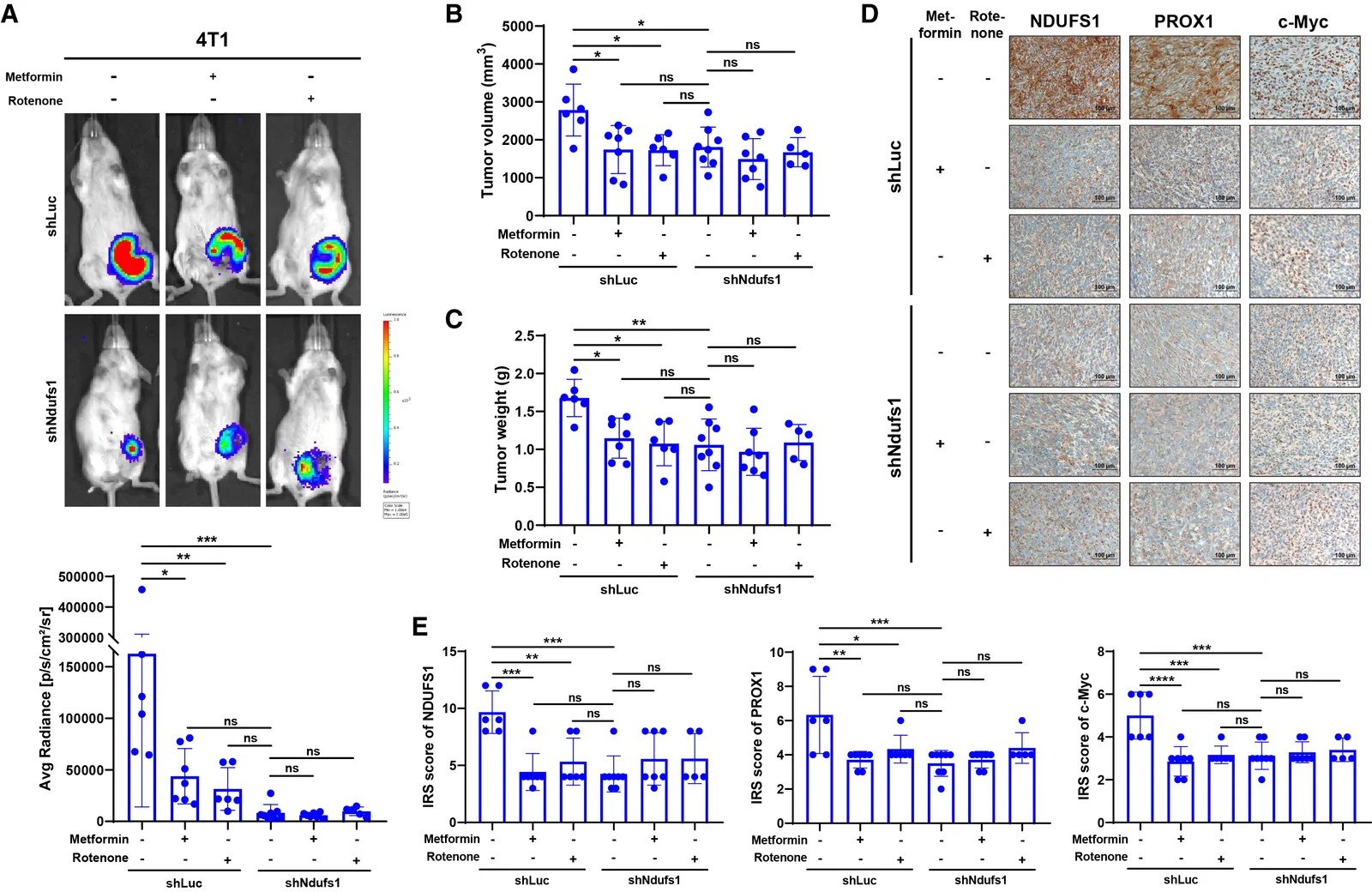
Mitochondrial complex I inhibition suppresses orthotopic breast tumor growth and tumoral expression of PROX1 and c-Myc in vivo (A) *In vivo* photon flux density emitted by luciferase-expressing 4T1 cells with or without Ndufs1 knockdown in BALB/c mice, which received metformin, rotenone, or vehicle treatment, detected and photographed by an IVIS 50 imaging system at the endpoint of the experiment. (B and C) Volume (B) and weight (C) of orthotopic tumors collected at the endpoint of the experiment. (D and E) Protein expression of NDUFS1, PROX1, and c-Myc in the orthotopic tumors, assessed using the immunoreactive score (IRS) method. Data are presented as mean ± SD; ∗, *p* < 0.05; ∗∗, *p* < 0.01; ∗∗∗, *p* < 0.001; and ∗∗∗∗, *p* < 0.0001; ns, not significant. shLuc, knockdown of firefly luciferase using shRNA; shNdufs1, knockdown of mouse Ndufs1 using shRNA clone D3.

## Discussion

This study provides new insights into the role of mitochondrial respiration—in particular, NDUFS1—in breast cancer progression. We found that NDUFS1, through the modulation of mitochondrial respiratory activity, positively regulates the malignant behaviors of breast cancer cells both *in vitro* and *in vivo* via the PROX1/c-Myc pathway (Figure 7).

**Figure 7:**
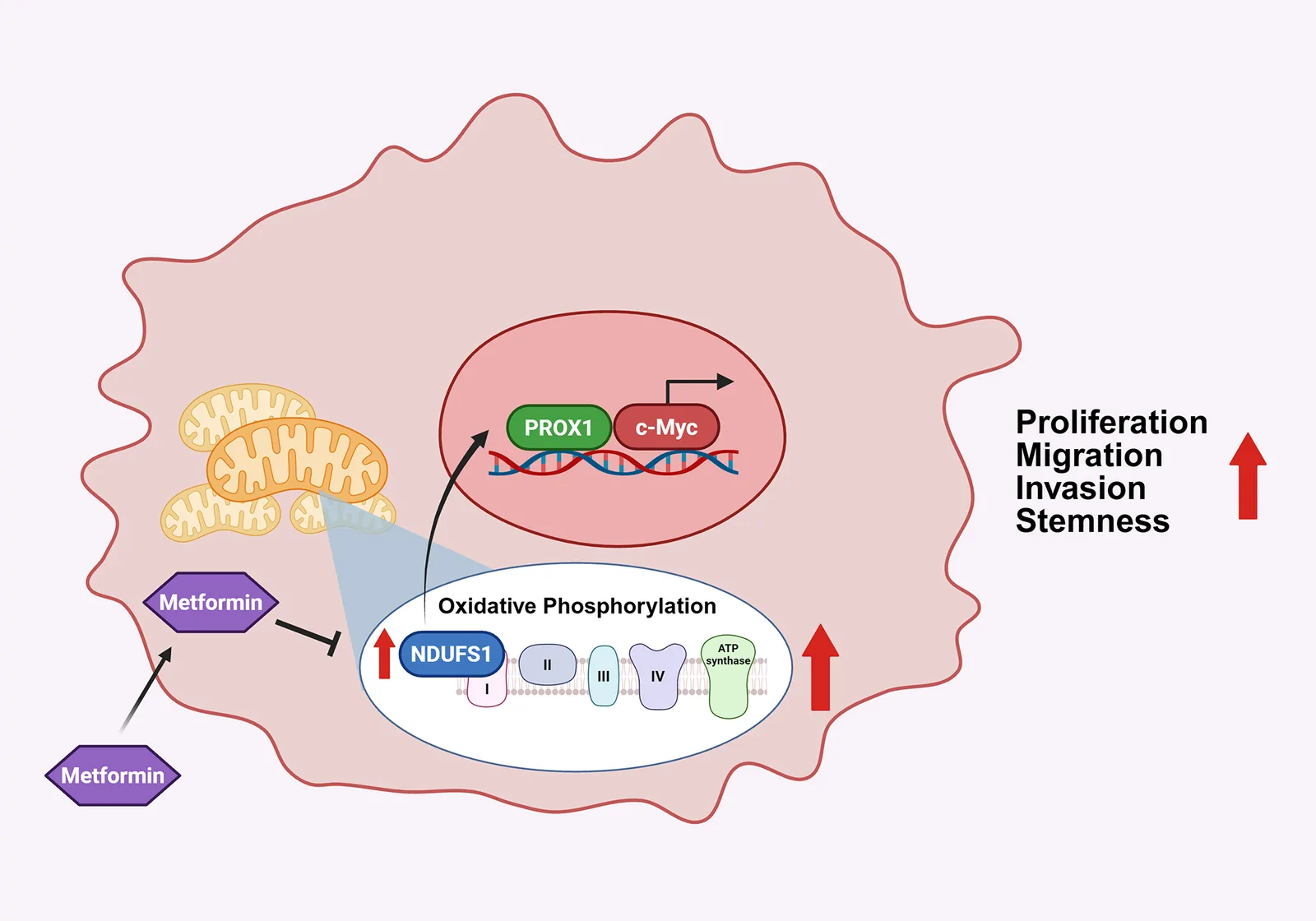
Schematic diagram of the signaling pathway mediating the pro-malignant effects of NDUFS1 on breast cancer cells based on this study NDUFS1 promotes oxidative phosphorylation and the upregulation of PROX1 and c-Myc, leading to increased proliferation, migration, invasion, and stemness-associated phenotypes in breast cancer cells. This signaling mechanism explains, at least in part, the enhanced progression of breast cancer in pathological conditions that promote mitochondrial respiration. It also highlights a vulnerability that can be targeted by metformin and other mitochondrial complex I inhibitors. See also Figure S8.

NDUFS1 promotes oxidative phosphorylation and the upregulation of PROX1 and c-Myc, leading to increased proliferation, migration, invasion, and stemness-associated phenotypes in breast cancer cells. This signaling mechanism explains, at least in part, the enhanced progression of breast cancer in pathological conditions that promote mitochondrial respiration (also see Figure S8). It also highlights a vulnerability that can be targeted by metformin and other mitochondrial complex I inhibitors.

Although low NDUFS1 expression has been associated with poorer clinical outcomes and cancer progression in renal cell, non-small cell lung and gastric carcinomas,^26^^,^^27^^,^^29^^,^^30^ we found that high NDUFS1 expression is significantly correlated with breast cancer progression (Figure 1; Table 1) and drives breast cancer cell malignant phenotypes (Figures 2 and 5), consistent with a prior study on SUM159 and MCF-7 cells.^35^ Our mechanistic investigation revealed that NDUFS1 promotes breast cancer cell aggressiveness via the enhancement of mitochondrial respiration and the PROX1/c-Myc pathway (Figures 3 and 4). Specifically, immunohistochemical analysis revealed a positive correlation between the expression of NDUFS1 and the expression of PROX1 and c-Myc in breast cancer tissues (Figures 2J and 4D), and regulation of NDUFS1 expression directly modulates c-Myc and PROX1 expression (Figures 2H, 4B, S2B, and S2C). Notably, c-Myc is downstream of PROX1 in the proposed pathway (Figures 4E and 4F). c-Myc has previously been reported to be highly expressed during breast cancer development,^43^^,^^44^ and bioinformatics analyses have shown an interaction between c-Myc and NDUFS1 in non-small cell lung cancer.^45^ Likewise, elevated PROX1 mRNA expression has also been reported in other cancers, including neuroblastoma, glioma, lung carcinoid tumor, small-cell lung carcinoma, colon cancer, hepatocellular carcinoma, and rhabdomyosarcoma.^46^ PROX1 has been found to drive the transition from benign to malignant phenotypes by altering cell polarity, extracellular matrix interactions, and cell adhesion,^47^ and to promote breast cancer cell invasion and metastasis via WNT/β-catenin signaling.^48^

Our findings show that the NDUFS1–PROX1–c-Myc signaling involves p21 as a downstream target, with NDUFS1, PROX1, and c-Myc negatively regulating p21 expression (Figures 4E, 4F, and S7D). c-Myc has been found to suppress p21 expression in MCF-7 cells,^41^ while PROX1 has also been found to negatively regulate p21 protein levels in hepatocellular carcinoma cells.^49^ Our study confirms these relationships and additionally identifies NDUFS1 as an upstream negative regulator of p21 expression. Downregulation of p21 in breast cancer cells has been shown to increase the proliferation and migration of breast cancer cells.^50^ Similarly, p21 knockdown contributes to epithelial-to-mesenchymal transition in breast epithelial cells.^51^ Our study thereby delineates an NDUFS1-PROX1-c-Myc-p21 signaling axis facilitating the tumor-promoting effects of NDUFS1 in breast cancer.

Cancer stemness, characterized by an increased capacity for self-renewal and enhanced motility of cancer cells, is strongly linked to tumor recurrence and metastasis in breast cancer.^52^^,^^53^ In the present study, we observed that NDUFS1 promoted cancer stemness-related properties in breast cancer cells, including proliferation and tumorsphere formation (Figures 2A, 2B, 2G, 5A, and 5D). Additionally, NDUFS1 positively modulated the expression of c-Myc in breast cancer cells (Figures 2H, 5E, S2B, and S2C). Since c-Myc is a transcription factor significantly positively associated with breast cancer stemness,^54^ our results suggest that NDUFS1 plays a key role in stemness-driven breast cancer progression.

Modification of cellular energy and redox status through mitochondrial metabolic reprogramming is strongly linked with cancer progression.^55^^,^^56^ Inhibition of mitochondrial complexes has emerged as a promising strategy for cancer treatment.^56^^,^^57^ Our *in vitro* results demonstrate that NDUFS1 promotes mitochondrial respiratory function (Figures 3A–3C and S3A), and treatment by metformin reversed the NDUFS1-induced increases in proliferation and migration of breast cancer cells (Figures 3D–3F), consistent with the anticancer effects of metformin observed in other studies.^10^ Notably, we found that metformin reversed the NDUFS1-induced increases in PROX1 and c-Myc expression in MDA-MB-231 cells (Figure 4G), indicating the important role of mitochondrial respiration in the NDUFS1–PROX1–c-Myc pathway.

How mitochondrial respiration specifically regulates PROX1 expression needs further investigation. Previous work demonstrated that deletion of mitochondrial complex III subunit QPC in lymphatic endothelial cells resulted in Prox1 downregulation, which was attributed to reduced citrate-induced hypoacetylation.^58^ This is also a plausible explanation for our study since loss of QPC results in decreased basal OCR,^59^ similar to the effect of NDUFS1 knockdown (Figures 3A and 3C), and OXPHOS deficiency can lead to reduced levels of citrate.^60^ Moreover, PROX1 histone acetylation at the H3K27ac mark has been shown to promote PROX1 expression.^61^ Collectively, these findings suggest that impaired mitochondrial respiration may suppress PROX1 expression through metabolic and epigenetic modifications.

Alternatively, NDUFS1-dependent changes in mitochondrial ROS production may influence PROX1 expression through redox-sensitive pathways, such as HIF-1α or Wnt/β-catenin signaling. However, the reported effects of these pathways on PROX1 expression are opposite to our observations. In particular, NDUFS1 downregulation or dysfunction has been associated with increased mitochondrial ROS production,^29^^,^^62^ which can stabilize HIF-1α,^29^ whereas HIF-1α has been reported to promote PROX1 expression.^63^^,^^64^ Likewise, ROS can activate the Wnt/β-catenin pathway,^65^ which has been shown to induce PROX1 expression in certain biological contexts via β-catenin/TCF signaling.^47^^,^^66^ Consequently, the reduced PROX1 expression observed following NDUFS1 knockdown in our study suggests that additional signaling or metabolic mechanisms may predominate over ROS-mediated pathways.

Ferroptosis is an iron-dependent programmed cell death characterized by lipid peroxidation and redox imbalance.^67^ Ferroptotic cell death has been shown to suppress tumor growth, making it an attractive therapeutic approach in breast cancer.^68^ Fe/S clusters, including those in NDUFS1, play an important role in different cellular processes, including iron homeostasis,^67^ and pharmacological perturbation of these clusters in cardiomyocytes by rotenone-induced ferroptosis.^69^ Similarly, mitochondrial complex I inhibitors BAY 87–2243^70^ and berberine^71^ also induced ferroptosis in melanoma and gastrointestinal cancer cells, respectively. These reports highlight the role of mitochondrial complex I, especially those subunits comprising Fe/S clusters, in inhibiting cell death by ferroptosis. It will be interesting to investigate in the future if the decrease in breast cancer cell proliferation and tumor growth following NDUFS1 knockdown or mitochondrial complex I inhibitor treatment also involved ferroptotic cell death.

It is worth noting that the effects induced by metformin, especially in the *in vitro* studies, may have been due to signaling cascades not specific to the PROX1–c-Myc axis. For instance, the antiproliferative effect of metformin could be due to a shift in ATP to adenosine monophosphate ratio, leading to cellular stress and subsequent AMPK-mTOR signaling.^72^ In mesenchymal stem-like/claudin-low cell lines, decreased cell growth, migration, and invasion can be attributed to metformin’s ability to block the activation of the TGF-β pathway.^73^ Invasive properties of MDA-MB-231 cells could also be inhibited by metformin via the downregulation of PGE2 levels.^74^ Consequently, inhibition of the AMPK-mTOR pathway and rescue of TGF-β- and PGE2-related signaling during metformin treatment may need to be performed in the future to ascertain the effect of metformin on PROX1/c-Myc-specific breast cancer cell behaviors.

In our study, metformin had significant effects on NDUFS1-overexpressing breast cancer cells, in contrast with negligible effects on the control cells, with short treatment times (Figures 3E, 3F, 4G, and S6). Such higher sensitivity of mitochondrial hypermetabolic cells to metformin was also observed in acute myeloid leukemia cells^75^ and thyroid cancer cells,^76^ and is consistent with observations in hypermetabolic, post-burn mice.^77^ NDUFS1 overexpression, which resulted in elevated mitochondrial respiration (Figures 3B and S3A) and increased cell proliferation, migration, and invasion (Figures 2B, 2D, and 2F), may have increased the reliance of breast cancer cells on mitochondrial OXPHOS, which enhanced their sensitivity to metformin.^72^^,^^78^Furthermore, MYC overexpression has been found to result in “glutamine addiction”, increased oxygen consumption, and a significant reduction in cell number upon treatment with metformin.^79^ Thus, the NDUFS1-induced increase in c-Myc protein level in our study (Figures S2B and S2C) may be an important mechanistic link between metformin and its enhanced anti-tumorigenic effects on the breast cancer cells.

The enhanced efficacy of metformin toward mitochondrial hypermetabolic breast cancer cells has positive implications in comorbidities that result in a metabolic shift toward OXPHOS, such as obesity. Recent studies show that obesity-associated signaling molecules lead to breast cancer progression and metabolic reprogramming toward mitochondrial respiration.^80^^,^^81^^,^^82^^,^^83^ Notably, we have shown that the obesity-associated adipocytokine resistin promotes breast cancer cell proliferation, migration, invasion, and stemness via direct stimulation^84^ or indirectly via the secretome of resistin-treated adipose-derived stem cells (ADSCs).^85^ To confirm that resistin also affects mitochondrial respiration in breast cancer cells, we co-cultured resistin-treated ADSCs with breast cancer cells and found a significant increase in the expression of NDUFS1 (Figure S8), suggesting that metformin may have therapeutic potential in obesity-associated breast cancer. Further *in vitro* and *in vivo* studies on the effects of metformin or rotenone on NDUFS1-overexpressing breast cancer cells, as well as those co-cultured with resistin-treated ADSCs, warrant future investigation.

Together, our findings support the increasingly important role of mitochondrial respiration—in particular, NDUFS1—in breast cancer cell malignancy. A NDUFS1–PROX1–c-Myc pathway may play a critical role in breast cancer progression and may additionally provide further basis for the use of metabolic drugs in breast cancer.

### Limitations of the study

While evidence of PROX1 and c-Myc modulation of p21 levels has been presented, direct confirmation of PROX1 and c-Myc regulation of breast cancer cell proliferation, migration, and invasion would further support the proposed signaling pathway. NDUFS1 regulation of PROX1 expression via OXPHOS deficiency-induced hypoacetylation and the crosstalk between PROX1 and c-Myc, such as the binding sites for PROX1 in the c-Myc promoter region for transcriptional activation, also deserve further investigation. Furthermore, apart from the effect of metformin and rotenone on breast tumor growth *in vivo*, a detailed *in vivo* study on the effect of these drugs on breast cancer metastasis would be highly beneficial. Finally, although a recent randomized controlled trial in 3,649 patients failed to find a positive benefit from metformin for invasive disease-free survival in breast cancer,^86^ our study provides evidence that may point toward the need for stratification of patients with higher NDUFS1 levels when designing future clinical trials.

## Resource availability

### Lead contact

Requests for further information and resources should be directed to and will be fulfilled by the lead contact, Shyng-Shiou F. Yuan (yuanssf@kmu.edu.tw).

### Materials availability

This study did not generate new unique reagents.

### Data and code availability

• Bulk RNA-seq data have been deposited at NCBI GEO as GEO: GSE328271 and are publicly available as of the date of publication.
• All data reported in this paper will be shared by the lead contact upon request.
• This paper does not report original code.
• Any additional information required to reanalyze the data reported in this paper is available from the lead contact upon request.

## Acknowledgments

The authors thank the Center for Laboratory Animals in Kaohsiung Medical University for the animal care and the Center for Research Resources and Development in Kaohsiung Medical University for providing the related services. This work was supported by grants from the National Science and Technology Council (NSTC 112-2314-B-037-112-MY3, NSTC 114-2314-B-037-098) and the Center for Intelligent Drug Systems and Smart Biodevices (IDS^2^B) from the Featured Areas Research Center Program within the framework of the Higher Education Sprout Project by the 10.13039/100010002Ministry of Education in Taiwan. This work was also supported by grants from 10.13039/501100011645Kaohsiung Medical University Hospital (KMUH111-1R37, KMUH112-2R42, KMUH113-3R37, KMUH114-4R44, KMUH-DK(A)113001, KMUH-DK(B)114007, KMUH-DK(B)114007-1) and 10.13039/501100004694Kaohsiung Medical University (KMU-DK(A)111005, KMU-DK(A)112006, KMU-DK(A)113002, KMU-DK(B)114007-1, KMU-TC112A03-5, KMU-TB114009-2 KT115P002), Taiwan.

## Author contributions

Conceptualization and methodology, Y.Y.W., P.Y.C., L.U.V., C.H.T., Y.K.C., S.C.S.H, S.L., M.F.H., C.H.W., Y.C.C., and S.S.F.Y.; investigation, Y.Y.W., P.Y.C., C.H.T., and Y.K.C.; formal analysis, Y.Y.W., C.H.T., M.F.H., and S.S.F.Y.; writing – original draft, Y.Y.W., L.U.V., and S.S.F.Y.; writing – review and editing, S.C.S.H. and S.L.

## Declaration of interests

The authors declare no competing interests.

## STAR★Methods

### Key resources table

**Table:** 

REAGENT or RESOURCE	SOURCE	IDENTIFIER
**Antibodies**		

Rabbit polyclonal anti-NDUFS1	GeneTex	Cat#GTX113787; RRID: AB_2037498
Rabbit polyclonal anti-PROX1	GeneTex	Cat#GTX129143; RRID: AB_2885908
Rabbit polyclonal anti-CD44	GeneTex	Cat#GTX102111; RRID: AB_1240596
Mouse monoclonal anti-Oct4 (clone GT486)	GeneTex	Cat#GTX627419; RRID: AB_11176459
Rabbit polyclonal anti-CD133 (C1C2, internal)	GeneTex	Cat#GTX100567; RRID: AB_1240577
Rabbit polyclonal anti-ALDH1A1	GeneTex	Cat#GTX123973; RRID: AB_11166810
Rabbit polyclonal anti-Nanog (N3C3)	GeneTex	Cat#GTX100863; RRID: AB_10615506
Rabbit polyclonal anti-ALDH2	GeneTex	Cat#GTX101429; RRID: AB_1949628
Rabbit polyclonal anti-SOX2 (N1C3)	GeneTex	Cat#GTX101507; RRID: AB_2038021
Rabbit polyclonal anti-MEF2C	GeneTex	Cat#GTX105433; RRID: AB_11173300
Rabbit polyclonal anti-GAPDH	GeneTex	Cat#GTX100118; RRID: AB_1080976
Rabbit polyclonal anti-alpha Tubulin	GeneTex	Cat#GTX112141; RRID: AB_10722892
Rabbit monoclonal anti-*c*-Myc (clone Y69)	Abcam	Cat#ab32072; RRID: AB_731658
Rabbit monoclonal anti-KLF4 (clone EPR3550(2)(ABC))	Abcam	Cat#ab151733; RRID: AB_2721027
Rabbit polyclonal anti-Wnt5a	Abcam	Cat#ab229200; RRID: AB_2890100
Rabbit monoclonal anti-Notch1 (clone D1E11)	Cell Signaling Technology	Cat#3608; RRID: AB_2153354

**Biological samples**		

Human breast tumor tissues	Kaohsiung Medical University Hospital (KMUH), Taiwan	KMUH Institutional Review Board approval nos. KMUHIRB-E(I)-20180136 and KMUHIRB-E(I)-20190424

**Chemicals, peptides, and recombinant proteins**		

XTT	Alfa Aesar	Cat#J61726
Phenazine methosulfate	Sigma-Aldrich	Cat#P9625
Matrigel	Corning	Cat#354234
Metformin	Sigma-Aldrich	Cat#D150959
Rotenone	Sigma-Aldrich	Cat#SI-R8875
Firefly D-luciferin	Biosynth	Cat#FL08608

**Critical commercial assays**		

Extracellular Oxygen Consumption Assay	Abcam	Cat#ab197243
Glycolysis Assay (Extracellular acidification)	Abcam	Cat#ab197244
Seahorse XF Cell Mito Stress Test Kit	Agilent Technologies	Cat#XFR103010-100
Bulk RNA sequencing service	Welgene Biotech	RNA Sequencing NGS Service

**Deposited data**		

TCGA TARGET GTEx, RSEM expected count (DESeq2 standardized)	UCSC Xena	https://toil-xena-hub.s3.us-east-1.amazonaws.com/download/TCGA-GTEx-TARGET-gene-exp-counts.deseq2-normalized.log2.gz
TCGA TARGET GTEx, TCGA TARGET GTEx selected phenotypes	UCSC Xena	https://toil-xena-hub.s3.us-east-1.amazonaws.com/download/TcgaTargetGTEX_phenotype.txt.gz
TCGA BRCA, phenotypes	UCSC Xena	https://tcga-xena-hub.s3.us-east-1.amazonaws.com/download/TCGA.BRCA.sampleMap%2FBRCA_clinicalMatrix
TCGA-BRCA (GDC), gene expression data	NCI Genomic Data Commons (via R: TCGAbiolinks)	Project: TCGA-BRCA; data category: Transcriptome Profiling; data type: Gene Expression Quantification; workflow type: STAR - Counts; sample type: Primary Tumor
TCGA-BRCA (GDC), clinical data	NCI Genomic Data Commons (via R: TCGAbiolinks)	Project: TCGA-BRCA; data category: Clinical; data type: Clinical Supplement; data format: BCR Biotab
TCGA-Clinical Data Resource (CDR) Outcome	NCI Genomic Data Commons; Liu et al.^87^	https://api.gdc.cancer.gov/data/1b5f413e-a8d1-4d10-92eb-7c4ae739ed81
Bulk RNA sequencing data of NDUFS1-knockdown and control MDA-MB-231 cells	This paper	GEO: GSE328271

**Experimental models: Cell lines**		

Human: MDA-MB-231 cells	Bioresource Collection and Research Center (BCRC), Hsinchu, Taiwan	BCRC#60425
Human: MCF-7 cells	BCRC	BCRC#60436
Human: T-47D cells	BCRC	BCRC#60250
Human: ZR-75-1 cells	BCRC	BCRC#60055
Human: Hs-578T cells	BCRC	BCRC#60120
Human: BT-549 cells	ATCC	ATCC#HTB-122
Mouse: 4T1 cells	ATCC	ATCC#CRL-2539

**Experimental models: Organisms/strains**		

Mouse: BALB/c, female, six weeks old	National Laboratory Animal Center, Taipei, Taiwan	Accession#RMRC11001

**Oligonucleotides**		

Human PROX1-targeting siRNA duplexes	Topgen	Product#DSSIRNA02
Negative control siRNA duplexes	Topgen	Product#USIRNAN01

**Recombinant DNA**		

Human NDUFS1-targeting shRNA lentiviral particles, pLKO.1_puro vector (clone G3)	National RNAi Core Facility, Academia Sinica, Taipei, Taiwan	ID: TRCN0000064631
Human NDUFS1-targeting shRNA lentiviral particles, pLKO.1_puro vector (clone H3)	National RNAi Core Facility	ID: TRCN0000064632
Mouse Ndufs1-targeting shRNA lentiviral particles, pLKO.1_puro vector (clone D3)	National RNAi Core Facility	ID: TRCN0000041564
Human MYC-targeting shRNA lentiviral particles, pLKO.1_puro vector	National RNAi Core Facility	ID: TRCN0000174055
Firefly luciferase-targeting shRNA lentiviral particles, pLKO.1_puro vector	National RNAi Core Facility	ID: TRCN000072249
Firefly luciferase-2A-GFP-expression lentiviral particles, CMV-Neo vector	GenTarget Inc	Cat#LVP403-PBS
Human NDUFS1-overexpression lentiviral particles, pReceiver vector	Topgen	ID: CMV63580201
Empty vector lentiviral particles, pReceiver vector	Topgen	ID: GMV63520201

**Software and algorithms**		

ImageJ	National Institutes of Health (NIH)	https://imagej.nih.gov/ij/
Prism	Graphpad Software	v9.5.0
SPSS	SPSS	v14.0
Oncomine	Rhodes et al.^88^	https://www.oncomine.org
Kaplan–Meier Plotter	Posta and Győrffy^89^	https://kmplot.com/analysis/
OncoDB	Tang et al.;^90^ Cho et al.^91^	https://oncodb.org/
R	R Core Team^92^	v4.5.3; https://www.r-project.org/
R package: rstatix	Kassambara^93^	v0.7.3; https://cran.r-project.org/package=rstatix
R package: TCGAbiolinks	Colaprico et al.^94^	v2.38.0; https://bioconductor.org/packages/release/bioc/html/TCGAbiolinks.html
R package: survival	Therneau;^95^ Therneau and Grambsch^96^	v3.8-6; https://cran.r-project.org/package=survival
R package: survminer	Kassambara et al.^97^	v0.5.2; https://cran.r-project.org/package=survminer

**Other**		

Transwell inserts, 8-μm pore size	Falcon, Corning	Cat#353097

### Experimental model and study participant details

#### Human samples

Breast tumor tissues were obtained from female breast cancer patients at Kaohsiung Medical University Hospital (KMUH), Taiwan, and confirmed using clinical and histological data from the Cancer Registry. This study was approved by the Institutional Review Board of KMUH (approval numbers KMUHIRB-E(I)-20180136 and KMUHIRB-E(I)-20190424), and patient informed consent was waived for the de-identified patient samples.

#### Cell lines

Human breast carcinoma cell lines MDA-MB-231, MCF-7, T-47D, ZR-75-1, and Hs-578T were purchased from the Bioresource Collection and Research Center (BCRC, Hsinchu, Taiwan), while the human breast carcinoma cell line BT-549 and the mouse breast carcinoma cell line 4T1 were purchased from the American Type Culture Collection (ATCC, Manassas, VA, USA). MDA-MB-231, MCF-7, T-47D, and BT-549 cells were maintained in Dulbecco’s Modified Eagle Medium (Gibco, Thermo Fisher Scientific, Waltham, MA, USA), while Hs-578T, ZR-75-1, and 4T1 cells were cultured in Roswell Park Memorial Institute 1640 (Gibco, Thermo Fisher Scientific) in a humidified incubator at 37 °C with 5% CO_2_. All culture media were supplemented with 10% fetal bovine serum (Biological Industries, Israel) and 1% penicillin/streptomycin/amphotericin B (Sartorius, Göttingen, Germany).

#### Animal models

Six-week-old female BALB/c mice were purchased from the National Laboratory Animal Center (NLAC, Taipei, Taiwan). Animal experiments were approved by the Institutional Animal Care and Utilization Committee (IACUC) of Kaohsiung Medical University (KMU), Kaohsiung, Taiwan (approval no. 108126). In accordance with the principles of the 3Rs, we minimized the number of animals used in our experiments.

### Method details

#### Gene knockdown and overexpression

To knock down NDUFS1 expression in MDA-MB-231, MCF-7, and 4T1 cell lines, lentiviral particles carrying a pLKO.1_puro vector (National RNAi Core Facility, Academia Sinica, Taipei, Taiwan) that expressing short hairpin RNA (shRNA) targeting human NDUFS1 (clone G3 (#1), ID: TRCN0000064631; clone H3 (#2), ID: TRCN0000064632) or mouse Ndufs1 (clone D3, ID: TRCN0000041564) were used, while a pLKO.1_puro vector expressing shRNA targeting firefly luciferase (ID: TRCN000072249; National RNAi Core Facility) was used as the negative control (see Figures S1 and S7). Lentiviral infection was achieved by adding the viral solution to cells in culture media containing 8 μg/mL polybrene. For selection, 2 μg/mL puromycin was added 48 hours after infection. Selected cells were cultured in media containing 1 μg/mL puromycin for the duration of the experiment.

To overexpress NDUFS1 in MDA-MB-231 and MCF-7 cell lines, ready-to-use lentiviral particles containing a pReceiver vector expressing human NDUFS1 were purchased from Topgen (Kaohsiung, Taiwan). For the negative control, lentiviral particles which carried an empty lentiviral vector (Topgen) were used. Lentiviral infection was performed by adding the viral solution to cells in culture media containing 8 μg/mL polybrene. After infection for 48 hours, 1 mg/mL G418 was added for selection. Selected cells were cultured in media containing 0.5 mg/mL G418 for the duration of the experiment.

For PROX1 knockdown, NDUFS1-overexpressing MDA-MB-231 cells were transfected with either small interfering RNA (siRNA) duplexes targeting human PROX1 (Topgen) or negative control siRNA duplexes (Topgen) using siRNA TopFect transfection reagent (Topgen) for 24 hours. For MYC knockdown, lentiviral particles carrying a pLKO.1_puro vector that expressed human MYC-targeting shRNA (ID: TRCN0000174055; National RNAi Core Facility) were used, with a pLKO.1_puro vector expressing firefly luciferase-targeting shRNA (National RNAi Core Facility) as the negative control. Lentiviral infection and selection were performed as described above for NDUFS1 knockdown.

#### XTT cell viability assay

Cells were seeded onto 96-well plates at a density of 4 × 10^3^ cells per well for MDA-MB-231 and MCF-7 cells, and 6×10^3^ cells per well for 4T1 cells. Cell viability was determined using XTT reagent with phenazine methosulfate according to the procedure described in a previous report.^98^

#### Transwell migration and invasion assays

Cells in 100 μL serum-free medium were seeded into 8-μm pore size Transwell inserts (Falcon, Corning, NY, USA), with or without Matrigel coating (Corning), for cell migration or invasion assay, respectively. The seeding densities for MDA-MB-231, MCF-7, and 4T1 cells were 2 × 10^4^, 4 × 10^4^, and 2 × 10^5^ cells per well, respectively, for the migration assay and 5 × 10^4^, 1 × 10^5^, and 2 × 10^5^ cells per well, respectively, for the invasion assay. The inserts were placed into the wells of a 24-well plate, with each well containing 500 μL serum-supplemented medium. The MDA-MB-231 and 4T1 cells were then incubated for 24 hours, while the MCF-7 cells were incubated for 48 hours (migration) or 72 hours (invasion). After incubation, the cells remaining on the upper side of the inserts were removed using cotton swabs, while the cells that migrated to the lower side of the inserts were fixed and stained with crystal violet (0.05%, Sigma-Aldrich, St. Louis, MO, USA) for 30 minutes. Images were captured using a light microscope (Nikon, Tokyo, Japan) and analyzed using ImageJ (https://imagej.nih.gov/ij/).

#### Tumorsphere formation assay

MCF-7 and 4T1 cells were seeded in ultra-low attachment 96-well plates (Corning) at a density of 500 cells per well with serum-free cell culture medium supplemented with 20 ng/mL recombinant human fibroblast growth factor basic, 20 ng/mL recombinant human epidermal growth factor, 10 μg/mL insulin, and 1× B-27. After 14 days of incubation, images of tumorspheres larger than 50 μm in diameter were captured using a light microscope (Nikon) and analyzed using ImageJ software (https://imagej.nih.gov/ij/).

#### Western blot

The detailed procedure was carried out according to a previous report.^98^ The primary antibodies used for western blot analysis included antibodies against NDUFS1 (1:1000, GTX113787, GeneTex, Irvine, CA, USA), c-Myc (1:1000, ab32072, Abcam, Cambridge, UK), PROX1 (1:1000, GTX129143, GeneTex), CD44 (1:1000, GTX102111, GeneTex), Oct4 (1:1000, GTX627419, GeneTex), CD133 (1:1000, GTX100567, GeneTex), ALDH1A1 (1:5000, GTX123973, GeneTex), Nanog (1:1000, GTX100863, GeneTex), KLF4 (1:1000, ab151733, Abcam), ALDH2 (1:1000, GTX101429, GeneTex), Notch1 (1:1000, #3608, Cell Signaling Technology, Danvers, MA, USA), SOX2 (1:3000, GTX101507, GeneTex), MEF2C (1:2500, GTX105433, GeneTex), Wnt-5a (1:250, ab229200, Abcam), GAPDH (1:60000, GTX100118, GeneTex), and α-Tubulin (1:10000, GTX112141, GeneTex).

#### Immunohistochemistry

For immunohistochemistry, slides were baked, de-waxed, and stained with avidin-biotin complexes following the procedure in our previous report.^99^ Immunohistochemical staining for NDUFS1 (1:100, GTX113787, GeneTex) and c-Myc (1:200, ab32072, Abcam) was carried out using the automated Bond-Max system, following the manufacturer’s instructions (Leica Microsystems, Wetzlar, Germany). For quantification, the immunoreactive score (IRS) was used to assess protein expression. IRS was calculated as the product of the percentage of stained cells and the intensity of staining. The score was evaluated independently by two experts under the same imaging conditions.

#### Oxygen consumption rate (OCR) measurements

OCR was measured using an Extracellular Oxygen Consumption Assay (ab197243, Abcam). MDA-MB-231 and MCF-7 cells were seeded in 96-well plates (black wall with clear flat bottom) at a density of 8 × 10^4^ cells per well and incubated overnight. The medium was replaced with fresh culture medium. After addition of the extracellular oxygen consumption reagent, the wells were promptly sealed with pre-warmed mineral oil. The extracellular oxygen consumption signal was measured using the CLARIOstar Plus plate reader (BMG LABTECH, Ortenberg, Germany) at 1.5-minute intervals for 120 minutes at Ex/Em = 360/650 nm. OCR was calculated as the slope of the time-course plot.

In order to quantify the different mitochondrial respiratory parameters, OCR was measured using Seahorse XFe24 Analyzer (Agilent Technologies, Santa Clara, CA, USA) coupled with Seahorse XF Cell Mito Stress Test Kit (Agilent Technologies). MDA-MB-231 and MCF-7 cells were seeded in 8-well Seahorse XF cell culture microplates at a density of 8 × 10^3^ cells per well and cultured in a humidified incubator at 37 °C with 5% CO_2_. The following mitochondrial modulators were administered in sequence according to the method described in a previous report: oligomycin, carbonyl cyanide-4-(trifluoromethoxy)phenylhydrazone (FCCP), and a mixture of rotenone and antimycin A. The OCR values were normalized by the cell number.^40^

#### Glycolysis assay

MDA-MB-231 and MCF-7 cells were seeded in 96-well plates at a density of 8 × 10^4^ cells per well and cultured overnight. The medium was replaced with fresh culture medium. After purging CO_2_ in a CO_2_-free incubator at 37°C with 95% humidity for 3 hours, the medium was replaced with the respiration buffer containing the Glycolysis Assay (Extracellular acidification) reagent (ab197244, Abcam). The glycolysis signal (lifetime signal) was measured using the CLARIOstar Plus plate reader (BMG LABTECH) at 1.5-minute intervals for >120 minutes at Ex/Em = 380/615 nm.

#### Bulk RNA sequencing (RNAseq) and gene overlap analysis

Exploratory analysis of gene regulation in NDUFS1-knockdown MDA-MB-231 cells was performed using a third-party bulk RNAseq service (Welgene Biotech, Taipei, Taiwan). Procedures for RNA sample preparation and library construction were carried out according to standard protocols of Illumina (San Diego, CA, USA). Paired-end sequencing (150PE) was performed on an a NovaSeq 6000 Sequencing System (Illumina), generating ≈89.7–99.2 million raw reads per sample. RNAseq reads were quality-trimmed and aligned to the reference genome (GRCh38) using Trimmomatic (v0.36)^100^ and HISAT2,^101^ respectively. Transcript assembly and expression quantification were performed using StringTie (v2.1.4).^102^^,^^103^ To identify candidate differentially expressed genes, a single biological replicate was used per condition (*n* = 1) and variance estimation was implemented using DESeq (v1.39.0)^104^ in “blind mode,” wherein all samples were treated as replicates.^105^ Candidate genes were determined by a *p*-value cut-off of 0.05 and a |log_2_FC| cutoff value of 1. These genes were annotated with gene ontology terms by performing functional enrichment analysis using clusterProfiler (v3.6).^106^ Gene ontology terms associated with proliferation, migration, and stemness were identified, and then the corresponding genes were pooled for gene overlap analysis. Real-time quantitative PCR was performed to confirm the RNAseq fold change of the shared genes (Figure S4).

#### Gene expression analysis using external datasets/platforms

Comparison of NDUFS1 gene expression in normal and breast cancer tissues was performed on the Oncomine platform^88^ (https://www.oncomine.org) using breast cancer microarray datasets from Curtis et al.^107^ and Ma et al.^108^ To perform subtype-specific NDUFS1 gene expression comparisons, analysis was performed in R^92^ (v4.5.3; R Foundation for Statistical Computing, Vienna, Austria) using the following datasets obtained from the University of California Santa Cruz (UCSC) Xena database (https://xenabrowser.net/datapages/): TCGA-TARGET-GTEx gene expression data (DESeq2-normalized RSEM expected counts), TCGA-TARGET-GTEx metadata (TCGA TARGET GTEx selected phenotypes), and PAM50 subtyping information from the TCGA-BRCA clinical data (TCGA BRCA phenotypes). NDUFS1 gene expression in normal breast tissues from the GTEx study and subtype-categorized breast primary tumor tissues from the TCGA study was compared using pairwise Wilcoxon rank-sum tests with Benjamini–Hochberg false discovery rate correction, implemented in the R package rstatix.^93^

Gene expression correlation analyses in the TCGA-BRCA cohort were performed on the OncoDB platform^90^^,^^91^ (https://oncodb.org/).

#### Survival analyses using external datasets/platforms

Survival analyses using publicly available breast cancer microarray (mRNA gene chip) datasets were performed on the Kaplan–Meier Plotter platform^89^ (https://kmplot.com/analysis/).

Additionally, overall survival analyses were carried out in R (v4.5.3; R Foundation for Statistical Computing) using the TCGA-BRCA dataset from the Genomic Data Commons (GDC) of the US National Cancer Institute (NCI), downloaded via the R package TCGAbiolinks.^94^ Gene expression (TPM), PAM50 subtype, and TNM stage data were obtained from the TCGA-BRCA (GDC) gene expression dataset, while the ER/PR/HER2 status was obtained from the TCGA-BRCA (GDC) clinical data. Age, tumor stage, and overall survival data were retrieved from the TCGA-Clinical Data Resource (CDR) Outcome.^87^ Analyses were restricted to female patients and primary tumor samples, excluding the PAM50 normal-like subtype. Tumor stages and TNM stages were consolidated into their primary categorical classifications. For patients with multiple samples, NDUFS1 expression values were averaged and clinical data were harmonized. The Kaplan–Meier plot was constructed using the surv_fit and ggsurvplot functions in the R package survminer,^97^ with NDUFS1 expression (TPM) as the stratification factor. The hazard ratio and the score test *p*-value (equivalent to the log-rank test in this setting) were calculated using a univariate Cox model (coxph) implemented in the R package survival.^95^^,^^96^ Multivariable Cox analysis of NDUFS1 was performed using two models with different covariate sets: (1) age, tumor stage, and PAM50 subtype; and (2) age, TNM stage, and ER/PR/HER2 status. NDUFS1 TPM values were log2-transformed without a pseudocount as all values were non-zero. Hazard ratios, 95% confidence intervals, and *p*-values were calculated using the coxph function in the R package survival.^95^^,^^96^

#### Animal study

Female BALB/c mice were randomized into two groups of ten. 4T1 cells (1 × 10^5^ cells in 50 μL normal saline and 50 μL Matrigel per mouse), transduced with lentiviral particles carrying a CMV-Neo vector expressing firefly luciferase-2A-GFP (GenTarget Inc, San Diego, CA, USA), were subcutaneously injected into the left fourth mammary fat pad of each mouse. The tumor size was measured weekly and calculated by the formula (width^2^ × length)/2. All mice were monitored weekly using an IVIS 50 *in vivo* imaging system (Xenogen, Alameda, CA, USA) following the injection of firefly D-luciferin (Biosynth, Staad, Switzerland). After six weeks, all mice were sacrificed, and the orthotopic tumors were collected for tumor weight and tumor volume measurements, followed by immunohistochemical analysis.

For the study of the therapeutic effects of metformin and rotenone, female BALB/c mice were randomly assigned to six groups, each consisting of eight mice. After the orthotopic tumor became measurable, injections of 1% DMSO, or 1% DMSO with 200 mg/kg metformin or 2.5 mg/kg rotenone were administered into the peritoneal cavity of the mice at a frequency of five times per week. After four weeks of treatment, all mice were sacrificed, and orthotopic tumors were collected for the assessment of tumor weight and volume, followed by immunohistochemical analysis.

### Quantification and statistical analysis

Experimental data were analyzed by the Student’s t-test for comparison between two groups and by one-way analysis of variance (ANOVA) with post-hoc Tukey’s test for comparison between multiple groups using Prism 9.5.0 software (GraphPad Software, Boston, MA, USA). Data are presented as mean ± SD, and *p*-values less than 0.05 are considered statistically significant.

Statistical analyses for the KMUH female breast cancer patient data were performed using the SPSS 14.0 statistical package (SPSS, Chicago, IL, USA). The cutoff point for high and low NDUFS1 level was determined by the receiver operating characteristic (ROC) curve. The associations between NDUFS1 level and clinicopathologic characteristics were analyzed by the Chi-square test.
