# Supplementary material for: NDUFS1 promotes malignant breast cancer behaviors through enhancement of mitochondrial respiration and PROX1/c-Myc signaling
Source: iScience. 2026 Oct 19;29(11):117942. doi: 10.1016/j.isci.2026.117942 (PMC13697328; doi:10.1016/j.isci.2026.117942)
Supplement: Supplementary file 1 [file mmc1.pdf]

iScience, Volume *29*

Supplemental information

NDUFS1 promotes malignant breast cancer behaviors through enhancement of mitochondrial respiration and PROX1/c-Myc signaling

Yen-Yun Wang, Pang-Yu Chen, Lester Uy Vinzons, Chih-Huang Tseng, Yuk-Kwan Chen, Stephen Chu-Sung Hu, Steven Lo, Ming-Feng Hou, Ching-Hu Wu, Yu-Chieh Chen, and Shyng-Shiou F. Yuan

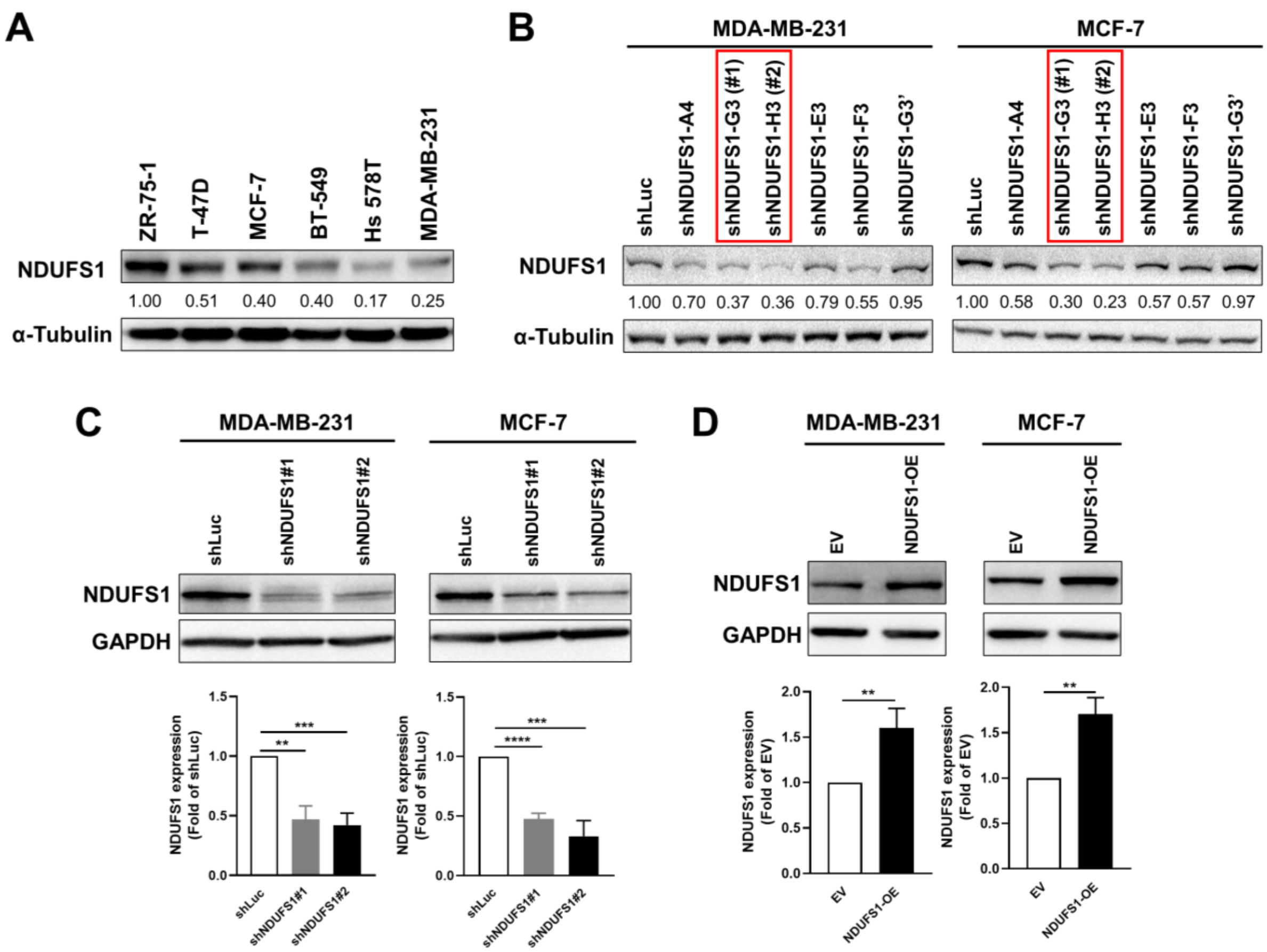

Figure S1. NDUFS1 knockdown and overexpression in human breast cancer cell lines, related to STAR Methods

(A) Western blot analysis of NDUFS1 protein expression in different human breast cancer cell lines.

(B) Representative results of western blots showing the knockdown efficiency of six shRNA clones targeting different consensus regions of human NDUFS1 in MDA-MB-231 and MCF7 cells. The numbers indicate the fold change of NDUFS1 protein expression relative to the control cells (shLuc group).

(C and D) Western blot analysis of NDUFS1 protein expression in MDA-MB-231 and MCF7 cells after NDUFS1 knockdown (C) and overexpression (D).

Data are presented as mean ± SD; **, $p < 0.01$; ***, $p < 0.001$; ****, $p < 0.0001$. shLuc, knockdown of firefly luciferase; shNDUFS1#1, knockdown of human NDUFS1 using shRNA clone G3 (#1); shNDUFS1#2, knockdown of human NDUFS1 using shRNA clone H3 (#2); EV, empty vector; NDUFS1-OE, overexpression of NDUFS1.

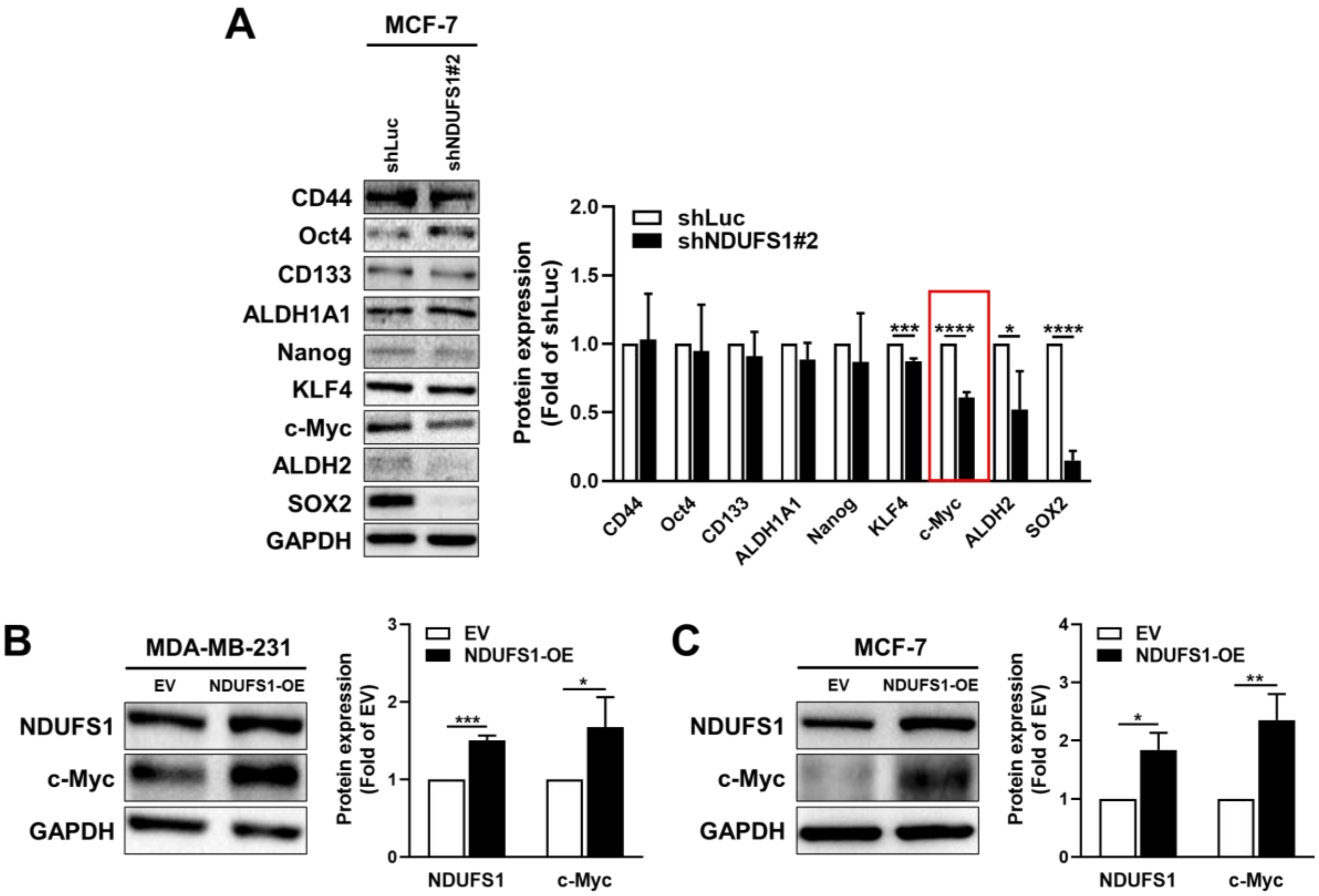

Figure S2. Stemness marker expression in breast cancer cells after NDUFS1 knockdown and overexpression, related to Figure 2

(A) Western blot analysis of the protein expression of different stemness markers in MCF-7 cells after NDUFS1 knockdown.

(B and C) Western blot analysis of NDUFS1 and c-Myc protein expression in MDA-MB-231 (B) and MCF-7 (C) cells after NDUFS1 overexpression.

Data are presented as mean ± SD; *, p < 0.05; **, p < 0.01; ***, p < 0.001; ****, p < 0.0001. shLuc, knockdown of firefly luciferase; shNDUFS1#2, knockdown of human NDUFS1 using shRNA clone 2; EV, empty vector; NDUFS1-OE, overexpression of NDUFS1.

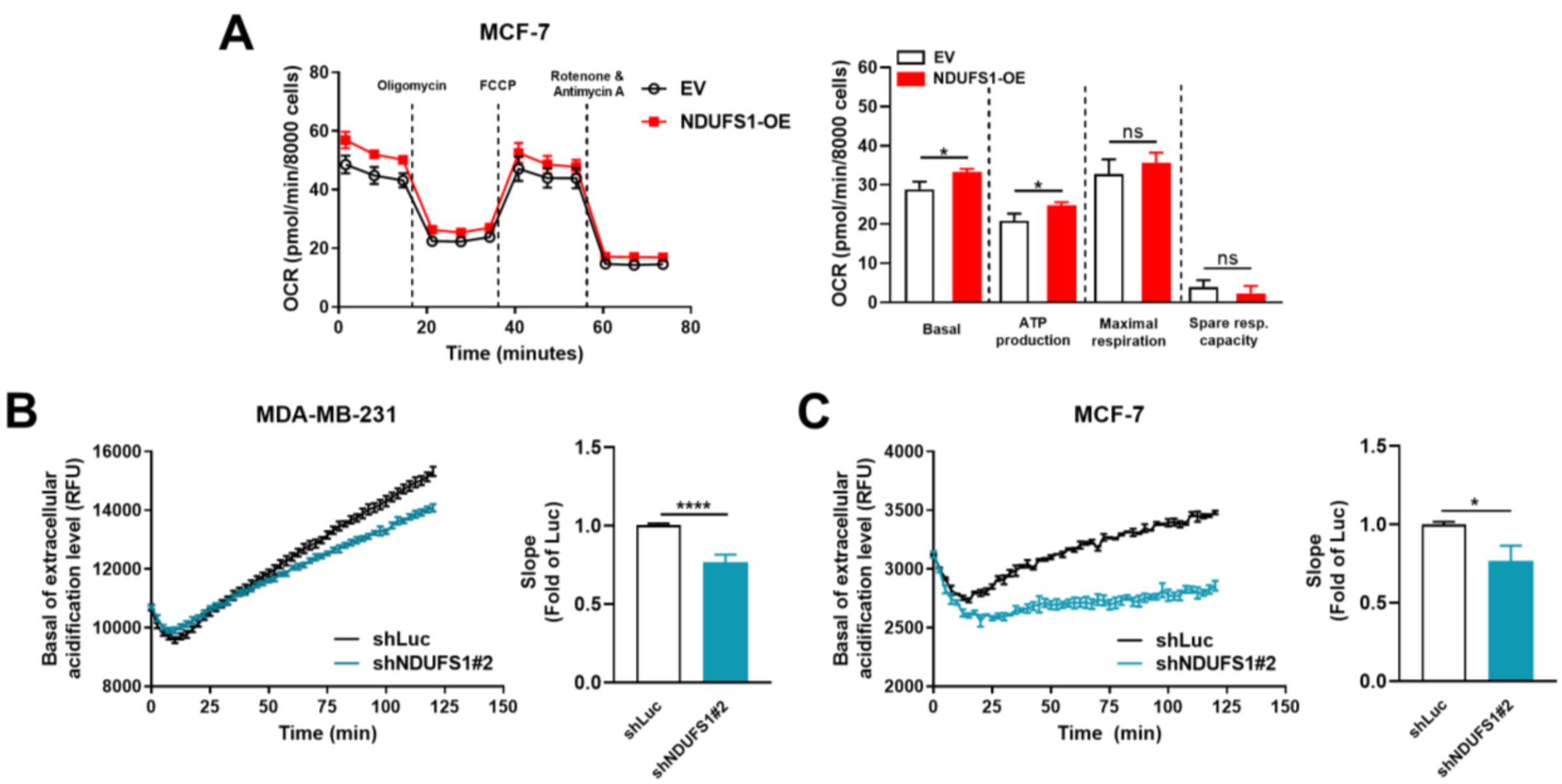

Figure S3. Mitochondrial respiration and extracellular acidification in breast cancer cells after modulation of NDUFS1 expression, related to Figure 3

(A) Mitochondrial respiratory parameters in MCF-7 cells after NDUFS1 overexpression, as measured by oxygen consumption rate (OCR) using Agilent Seahorse XFe24 Analyzer via sequential delivery of the indicated mitochondrial modulators.

(B and C) Basal extracellular acidification in MDA-MB-231 (B) and MCF-7 (C) cells after NDUFS1 knockdown, with the slope (ΔRFU/Δmin) being the extracellular acidification rate (ECAR).

Data are presented as mean ± SD; *, $p < 0.05$; ****, $p < 0.0001$; ns, not significant. shLuc, knockdown of firefly luciferase; shNDUFS1#2, knockdown of human NDUFS1 using shRNA clone 2; EV, empty vector; NDUFS1-OE, overexpression of NDUFS1.

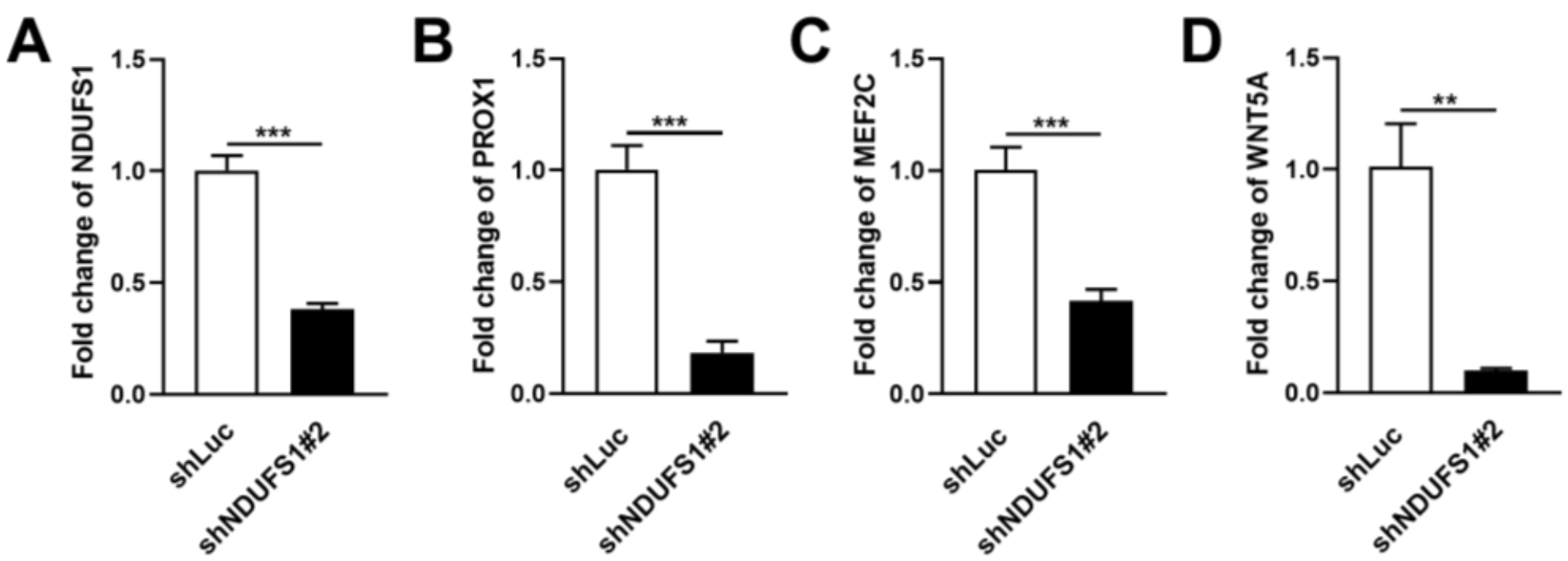

Figure S4. Real-time quantitative PCR measurement of mRNA levels in MDA-MB-231 cells after NDUFS1 knockdown, related to Figure 4

Relative mRNA expression of NDUFS1 (A), PROX1 (B), MEF2C (C), and WNT5A (D) in MDA-MB-231 cells after NDUFS1 knockdown, as determined by real-time quantitative PCR. Data are presented as mean ± SD; **, $p < 0.01$; ***, $p < 0.001$. shLuc, knockdown of firefly luciferase; shNDUFS1#2, knockdown of human NDUFS1 using shRNA clone 2.

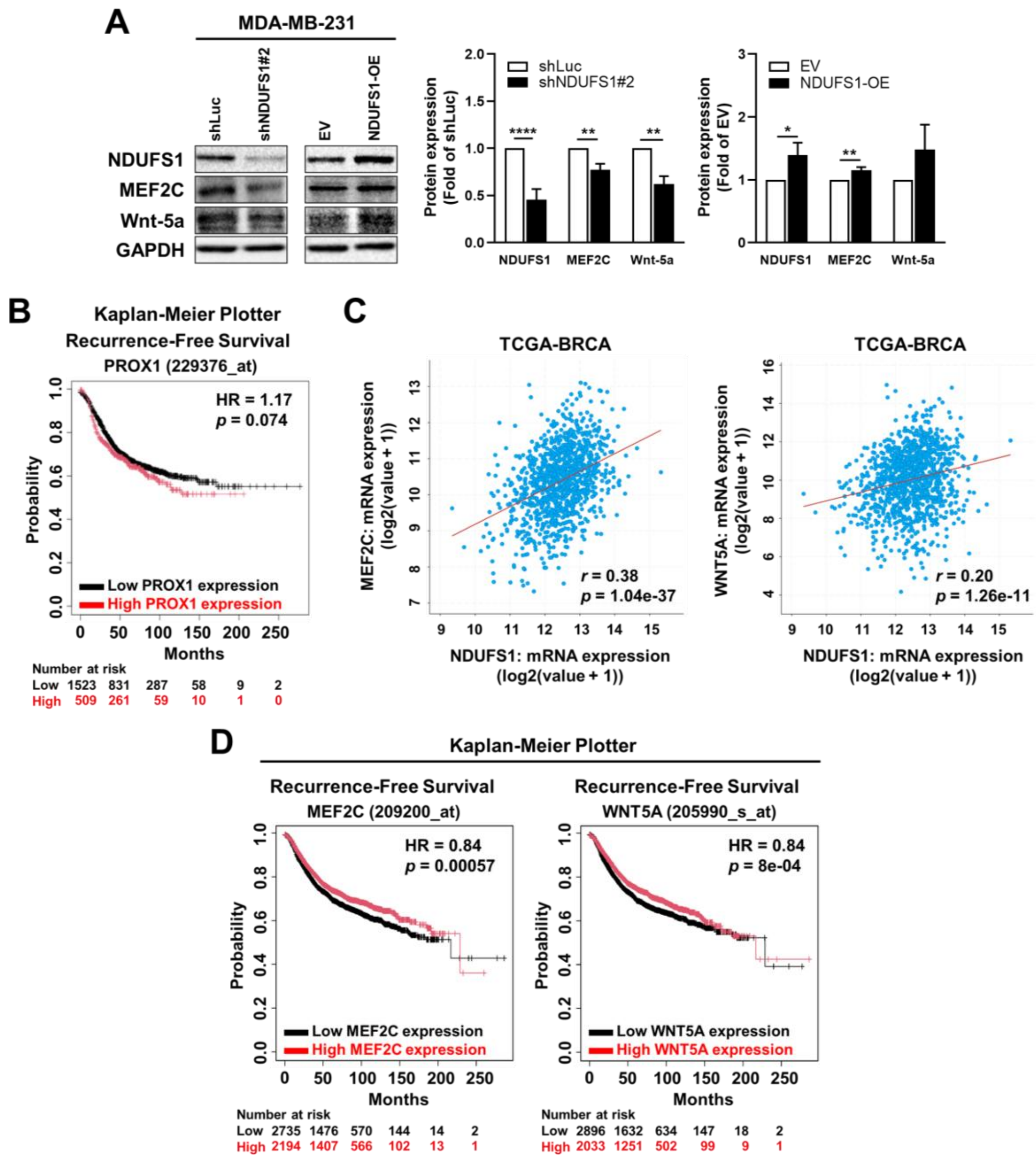

Figure S5. Western blot, survival, and correlation analysis of key NDUFS1-regulated genes/proteins in the study, related to Figure 4

(A) Western blot analysis of NDUFS1, MEF2C, and Wnt-5a protein expression in MDA-MB-231 cells after NDUFS1 knockdown and overexpression. Data are presented as mean ± SD; *, $p < 0.05$; **, $p < 0.01$; ****, $p < 0.0001$. shLuc, knockdown of firefly luciferase; shNDUFS1#2, knockdown of human NDUFS1 using shRNA clone 2; EV, empty vector; NDUFS1-OE, overexpression of NDUFS1.

(B) Kaplan–Meier graph of recurrence-free survival from the Kaplan–Meier Plotter (https://kmplot.com/analysis/), using publicly available breast cancer microarray datasets, stratified according to PROX1 mRNA expression.

(C) Correlation between NDUFS1 mRNA expression and MEF2C and WNT5A mRNA expression in the TCGA-BRCA dataset.

(D) Kaplan–Meier graphs of recurrence-free survival from the Kaplan–Meier Plotter, using publicly available breast cancer microarray datasets, stratified according to MEF2C and WNT5A mRNA expression.

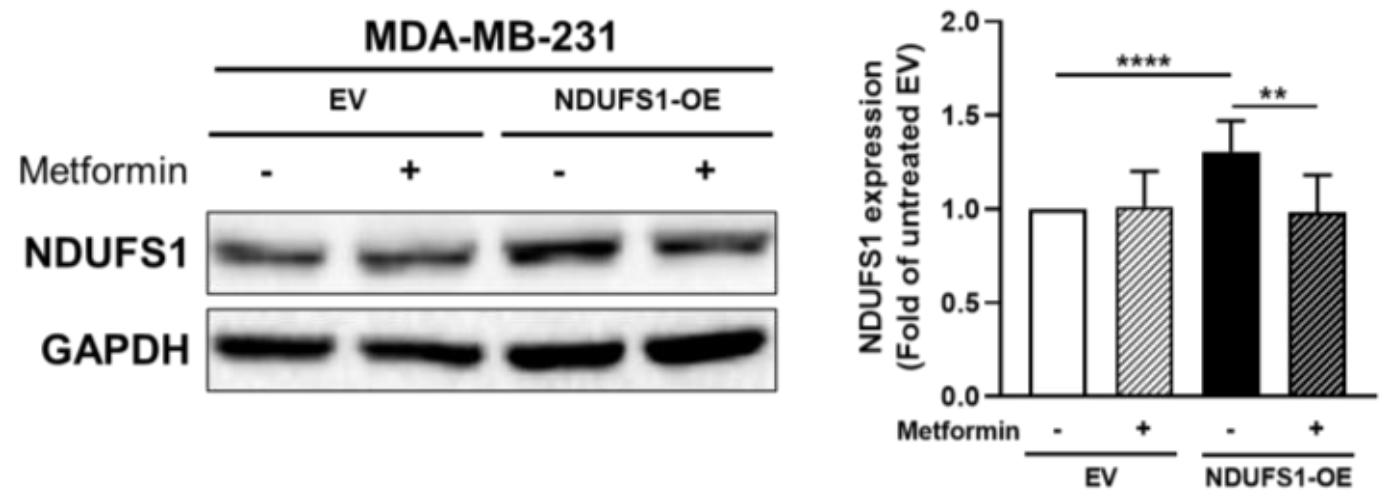

Figure S6. NDUFS1 protein expression in MDA-MB-231 cells after metformin treatment, related to Figure 4

Western blot analysis of NDUFS1 protein expression in MDA-MB-231 cells with or without NDUFS1 overexpression after metformin (30 mM) treatment for 24 hours. Data are presented as mean ± SD; **, $p < 0.01$; ****, $p < 0.0001$. EV, empty vector; NDUFS1-OE, overexpression of NDUFS1.

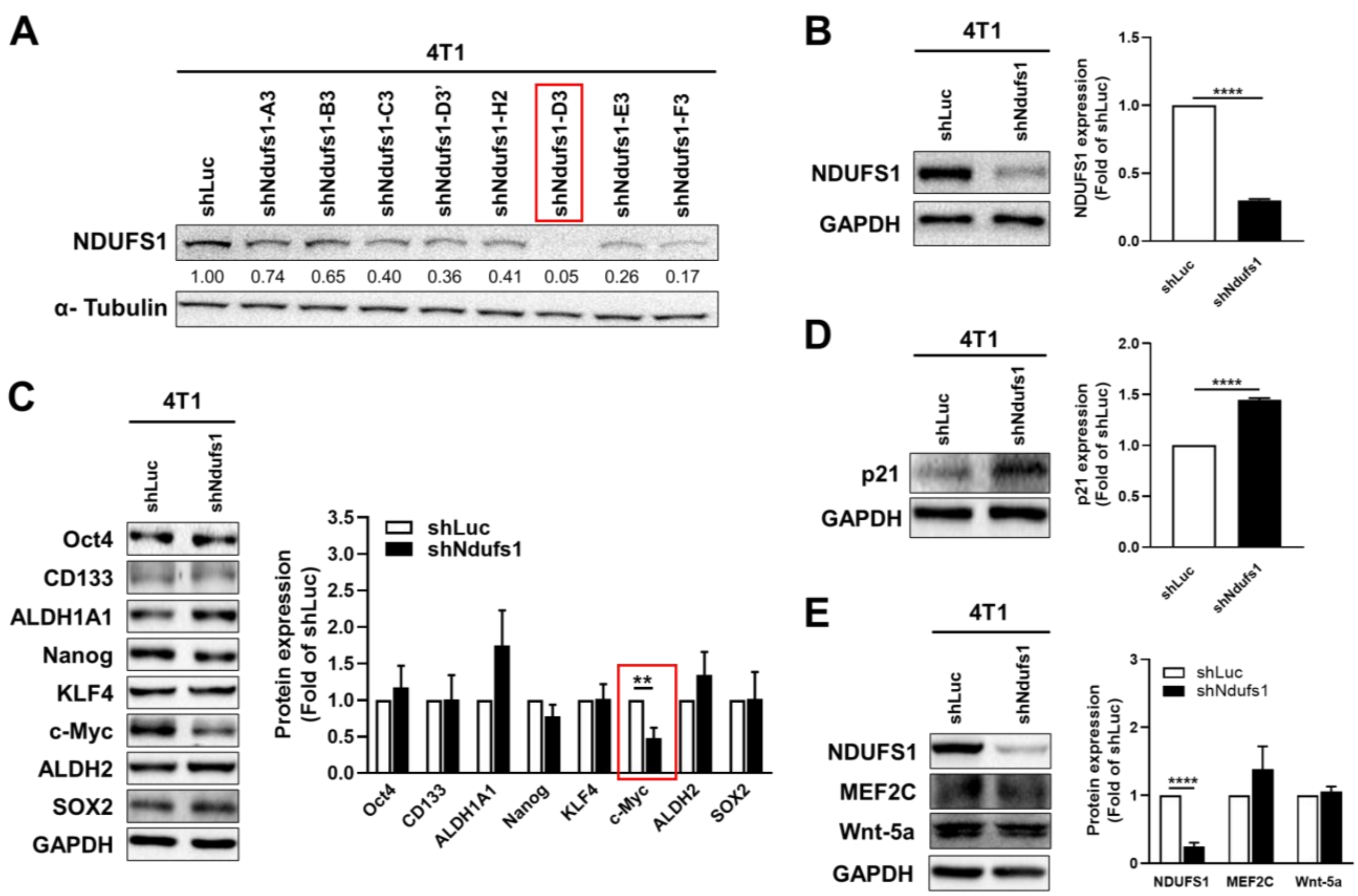

Figure S7. NDUFS1 knockdown and the resulting stemness marker, p21, MEF2C, and Wnt-5a protein expression in 4T1 cells, related to Figure 5 and STAR Methods

(A) Representative results of western blot showing the knockdown efficiency of eight shRNA clones targeting different consensus regions of mouse Ndufs1 in 4T1 cells. The numbers indicate the fold change of NDUFS1 protein expression relative to the control cells (shLuc group).

(B–E) Western blot analysis of NDUFS1 (B), stemness marker (C), p21 (D), and MEF2C and Wnt-5a (E) protein expression in 4T1 cells after Ndufs1 knockdown.

Data are presented as mean ± SD; **, $p < 0.01$; ****, $p < 0.0001$. shLuc, knockdown of firefly luciferase; shNdufs1, knockdown of mouse Ndufs1 using shRNA clone D3.

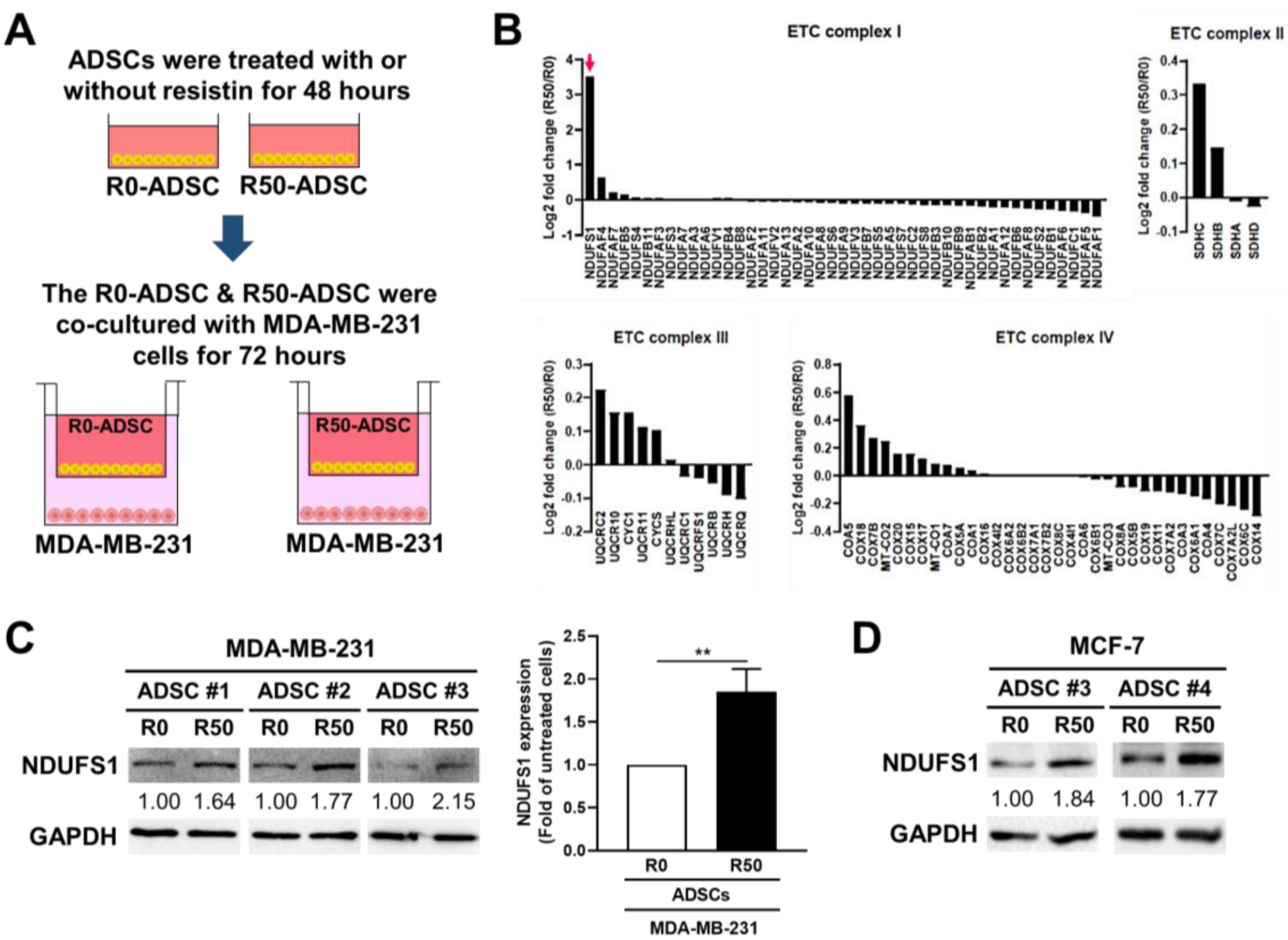

Figure S8. NDUFS1 expression in breast cancer cells after co-culture with resistin-treated adipose-derived stem cells, related to Figure 7

(A) Adipose-derived stem cells (ADSCs) were incubated in the presence (R50-ADSC) or absence (R0-ADSC) of resistin (50 ng/mL) for 48 hours. The ADSCs were co-cultured with MDA-MB-231 or MCF-7 cells for 72 hours in a 6-well plate using a Transwell insert (0.4 µm pores, Costar, Corning, NY, USA), with the ADSCs seeded in the upper chamber and the MDA-MB-231 cells seeded in the lower chamber.

(B) Fold change in the expression of genes related to mitochondrial electron transport chain (ETC) complexes I, II, III, and IV in MDA-MB-231 cells co-cultured with R50-ADSCs, relative to the gene expression in MDA-MB-231 cells co-cultured with R0-ADSCs, according to bulk RNA sequencing analysis.

(C and D) Western blot analysis of NDUFS1 protein expression in MDA-MB-231 (C) and MCF-7 (D) cells after co-culture with R0-ADSCs and R50-ADSCs. Data are presented as mean ± SD; **, $p < 0.01$.

Table S1. Multivariable Cox overall survival analysis of NDUFS1 expression in TCGA-BRCA (GDC) using two different sets of covariates, related to Figure 1

Model	Variable	HR	95% CI	*p*-value
NDUFS1-age-stage-PAM50	NDUFS1 expression[a]	1.44	1.10–1.90	**0.009**[c]
	Age[b]	1.04	1.03–1.06	<0.0001
	Tumor stage (vs. Stage I)			
	Stage II	1.89	1.05–3.41	0.03
	Stage III	4.07	2.20–7.52	<0.0001
	Stage IV	12.90	5.93–28.08	<0.0001
	PAM50 subtype (vs. Luminal A)			
	Luminal B	1.26	0.81–1.98	0.30
	HER2	2.68	1.54–4.64	0.0005
	Basal	1.83	1.13–2.96	0.01
NDUFS1-age-TNM-receptor	NDUFS1 expression[a]	1.99	1.26–3.14	**0.003**[c]
	Age[b]	1.05	1.03–1.08	<0.0001
	T stage (vs. T1)			
	T2	0.88	0.43–1.81	0.73
	T3	2.11	0.85–5.21	0.11
	T4	2.25	0.67–7.58	0.19
	N stage (vs. N0)			
	N1	1.00	0.50–2.01	0.99
	N2	1.62	0.65–4.03	0.30
	N3	3.31	1.19–9.20	0.02
	M stage (vs. M0)			
	M1	7.70	2.86–20.72	<0.0001
	ER status (vs. Positive)			
	Negative	2.92	1.08–7.90	0.03
	PR status (vs. Positive)			
	Negative	0.90	0.36–2.26	0.82
	HER2 status (vs. Negative)			
	Positive	1.34	0.70–2.58	0.38

HR, hazard ratio; CI, confidence interval; PAM50, Prediction Analysis of Microarray 50; T, primary tumor size; N, regional lymph node involvement; M, distant metastasis status; ER, estrogen receptor; PR, progesterone receptor; HER2, human epidermal growth factor receptor 2

[a]NDUFS1 gene expression values are log2-transformed transcript per million (TPM) values and were analyzed as a continuous variable; HR represents the change in risk per two-fold increase in NDUFS1 expression (TPM).

[b]Age refers to the age at initial pathologic diagnosis and was analyzed as a continuous variable.

[c]Indicates that NDUFS1 expression is an independent prognostic marker in this model. A model concordance index of 0.775 and 0.815 was obtained for the NDUFS1-age-stage-PAM50 model and the NDUFS1-age-TNM-receptor model, respectively.
